# Natural Deep Eutectic Solvent as Extraction Media for the Main Phenolic Compounds from Olive Oil Processing Wastes

**DOI:** 10.3390/antiox9060513

**Published:** 2020-06-11

**Authors:** Sonia Bonacci, Maria Luisa Di Gioia, Paola Costanzo, Loredana Maiuolo, Sofia Tallarico, Monica Nardi

**Affiliations:** 1 Dipartimento di Scienze della Salute, (Università Magna Græcia, Viale Europa, 88100-Germaneto CZ), Italy; s.bonacci@unicz.it (S.B.); s.tallarico@unicz.it (S.T.); monica.nardi@unicz.it (M.N.); 2 Dipartimento di Farmacia e Scienze della Salute e della Nutrizione, Edificio Polifunzionale, Università della Calabria, 87030 Arcavacata di Rende, Cosenza; 3 Dipartimento di Chimica e Tecnologie Chimiche, Università della Calabria, Cubo 12C, 87036-Arcavacata di Rende (CS), Italy; loredana.maiuolo@unical.it

**Keywords:** natural deep eutectic solvents, microwave assisted extraction, phenolic compounds, olive oil processing wastes

## Abstract

In this new century, sustainable development challenges chemical sciences to develop new and clean technological processes. The agri-food industry produces significant quantities of waste, raising significant economic and environmental concerns. Food waste valorization using environmentally friendly procedures is of increasing importance. This study describes the use of several Natural Deep Eutectic Solvents (NADESs) for the microwave-assisted extraction (MAE) of valuable bioactive phenolic compounds from olive oil processing wastes. The extracted samples were characterized by liquid chromatography electrospray ionization quadrupole time-of-flight mass spectrometry (LC-ESI-QTOF/MS) analysis and the quantification of the phenolic compounds was performed by HPLC analysis. The obtained data were compared with those obtained using water as the solvent in the same extraction conditions. The extraction process is nontoxic, simple and selective and meets most of the criteria to be considered as a sustainable process, with the solvents arising directly from nature.

## 1. Introduction

Every year, the olive oil processing industry produces a great amount of waste, such as twigs, leaves and olive mill wastewater (OMW). The low cost of these kinds of residues favors their application in various processes, varying from use in animal feed or in nutraceutical, pharmaceutical and cosmetic manufacturing. Thus, olive leaves are gaining increasing interest for their use in high-added-value compounds.

It is well established that the leaves of *Olea europaea* L. contain a quantity of phenolic compounds higher than that present in the fruit or in virgin olive oil: 1450 mg of total phenols/100 g of fresh leaves [1] against 110 mg/100 g of fruit [2] and 23 mg/100 mL of extra virgin olive oil [3].

In particular, olive leaves have phenolic compounds in common with other plants, but represent a rich source of secondary metabolites, among which secoiridoids and flavonoids stand out. Olive leaves are rich in secoiridoids, especially oleosides, which are specific secoiridoids of the olive trees commonly esterified at the phenolic portion. Oleuropein (Ole), the most abundant bioactive phenol in olive leaf extract, and ligstroside are the secoiridoid glucosides that confer organoleptic properties to olive leaves and fruits. The scientific literature available on olive polyphenols is focused mainly on Oleuropein, thanks to its important pharmacological activities [4,5,6,7,8].

The hydrolysis of oleuropein and ligstroside results in the formation a series of degradation products, all of them less hydrophilic than the original secoiridoids [9,10,11], and therefore more soluble in the oily matrix. In recent years, the biological properties of aglycone forms have been studied [12], in particular oleocanthal (*p*-HPEA-EDA), the elenolic acid ester of the tyrosol responsible of pharyngeal pungency sensation that occurs when consuming extra virgin olive oil (EVOO) [13], and its hydroxytyrosol derivative analog oleacein (3,4-DHPEA-EDA) [14,15].

After Beauchamp et al. first described the anti-inflammatory properties of oleocanthal as being similar to ibuprofen [14], numerous pharmacological/biological activities were reported for this olive oil secoiridoid derivative and its analog oleacein [15,16,17,18,19,20,21]. Unfortunately, only small amounts of either secoiridoid can be obtained from natural matrices (fruits and leaves of the olive tree, OMW, EVOO, etc.), which has prompted researchers to develop different pathways for total and semi-synthesis of oleacin and oleocanthal [22,23,24,25,26].

Hence, the many chemical, agronomical and pharmaceutical positive characteristics of oleuropein, and the possibility of synthesizing the most valuable secoiridoid derivatives starting from this compound alone, together contributed to increasing the interest in the recovery of phenolic compounds from olive leaves (waste product of olive oil industries) with the aim of producing functional foods and nutraceuticals.

Conventionally, the extraction of phenolic compounds from plant leaves and fruits is carried out by maceration, using organic solvents such as ethanol, methanol, dichloromethane, acetone, hexane and ethyl acetate [27]. The yields using these solvents are high, and the product obtained is of good quality. However, these processes require long treatment times and high temperatures, and the solvents used are harmful to human health and the environment; additionally, the extracts, prior to use, must be subjected to solvent removal and purification treatments.

Currently, the development of innovative and environmentally friendly extraction procedures is in increasing demand, as this will be extremely valuable in terms of reducing both the extraction time and the solvent consumption through the application of profitable strategies that are in line with the principles of Green Chemistry [28,29,30].

In recent years, the concepts of the green economy and eco-sustainability have led to increased attention being paid to deep eutectic solvents (DESs), new solvents that are emerging as a promising and greener alternative to conventional organic solvents [31,32,33,34,35]. DESs represent a new generation of solvents that are mostly based on mixtures of cheap and easily available components: non-toxic quaternary ammonium salts (e.g., choline chloride) and an unbound, naturally derived hydrogen donor (e.g., amides, sugars, alcohols, polyols) [36].

Moreover, the mechanism by which some living beings (beetles, frogs, earthworm, tardigrades, stoneflies, etc.) are able to survive the enormous temperature deviations of some of the most extreme habitats on our planet has recently been discovered. They are able to produce metabolites that, through a process known as vitrification, prevent the crystallization of water, which would lead to cell destruction. These compounds, mainly sugars, organic acids, choline derivatives, or urea, when combined in a certain composition, form a new deep eutectic liquid phase referred to as Natural Deep Eutectic Solvents (NADES) [37].

NADES fully respect the principles of Green Chemistry and are considered “future solvents”, because they allow nutraceuticals and food-grade extracts to be obtained [38,39]. One of the most important advantages of NADES is the possibility of easily preparing these solvents, together with the large number of combinations that could be applied [40].

Considering the numerous structural possibilities of NADES and the possibility of designing their physical-chemical properties in order to adapt them to different purposes, they can without any doubt be considered “design solvents”. Currently, the use of DES as extraction solvents for phenolic compounds and more generally for the extraction of natural products for pharmaceutical applications is by far the most studied application [41].

**Figure 1:**
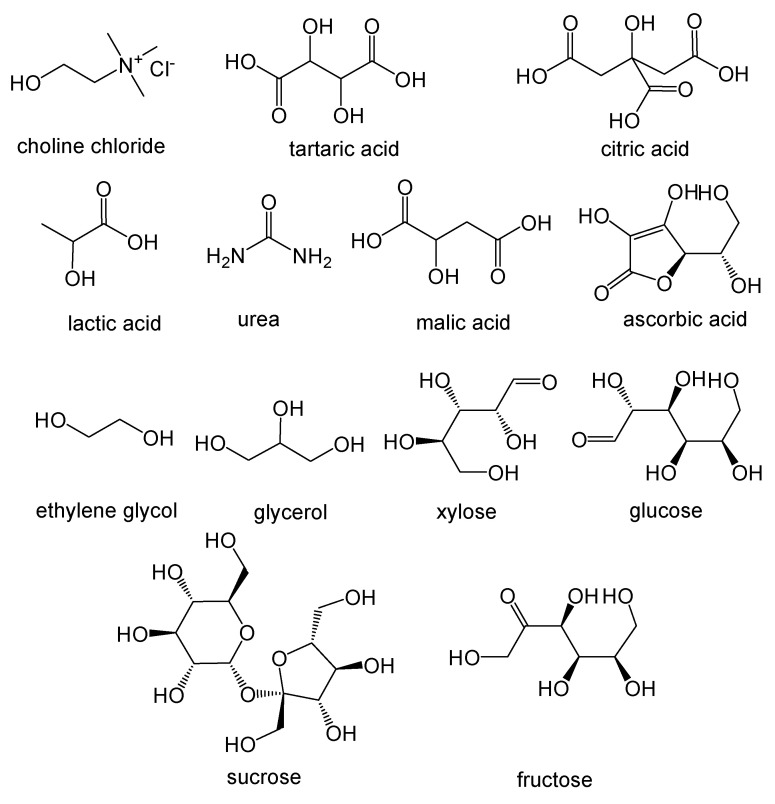
Chemical structure of some compounds that can be constituents of Natural Deep Eutectic Solvents.

A large number of NADES based on natural compounds (Figure 1), in particular primary metabolites such as sugars, organic acids, urea and quaternary ammonium salts such as choline chloride, have been reported and tested [31,42].

Some authors have studied DES-assisted extraction of phenolic compounds, demonstrating that many compounds are dissolved better than in other traditional and alternative solvents [43,44,45,46]. In particular, DES have the ability to donaate and accept protons and electrons, which gives them the ability to form hydrogen bonds, thus increasing their dissolution capacity. Therefore, since microwave-assisted extraction (MAE) has emerged in recent years on account of its numerous advantages (shorter extraction times, reduced solvent consumption, and more effective extraction), we planned to develop a green methodology that envisaged the combined action of some NADESs and the activation of microwaves for the extraction of phenolic compounds from olive leaves and ripe drupes coming from the production of the olive oil.

## 2. Materials and Methods

### 2.1. Samples, Standards, and Reagents

The olive leaves (Coratina cultivar, *Olea europaea* L.) and the ripened olive drupes (Leccino cultivar) used in this study were provided by CREA-Research center (Rende, CS, (Italy). Samples were randomly collected from several trees and immediately transferred to the laboratory, washed with distilled water, and dried under controlled temperature, for 48 h at 50 °C, until a constant weight was achieved. The ripened olive drupes were ground and stored at −20 °C until extraction.

### 2.2. NADEs Preparation

NADESs were fundamentally prepared by heating choline chloride with different hydrogen donors. The components were placed in a round-bottom flask and heated to 80 °C in a water bath with agitation until a homogeneous liquid was formed. Additionally, an addition of 20% *v/v* water in the NADES solutions was performed.

### 2.3. Extraction of Phenolic Compounds from Olive Leaves or Ripened Olive Drupes with NADES

2 g of sample was mixed with 5 mL of NADES in a closed-vessel and the mixture was subjected to microwave (MAE) at 100 W (time 10 or 30 min at 80 °C). The obtained extract was centrifuged at 1000 rpm for 10 min and the supernatant was collected. The sample was then filtered under vacuum, diluted with ethanol for the successive analysis by HPLC and LC-ESI-QTOF/MS.

### 2.4. Extraction of Phenolic Compounds from Olive Leaves or Ripened Olive Drupes with Water (Control Sample)

In parallel, with the aim of comparing the extraction efficiency exhibited by the NADES, the same procedure was carried out using water as the solvent.

### 2.5. HPLC Analysis

HPLC analysis was performed using Thermo Scientific (Rodano, MI, Italy) Dionex Ultimate 3000, equipped with a 25 cm × 4.6 mm Thermo Scientific Hypersil GOLD C18 column packed with 5 μm particles. For HPLC separation of the phenolic compounds in DESs, a gradient elution with a mixture of solvents A (H_2_O/trifluoroacetic acid, pH = 2.46) and B (acetonitrile) was used. The column was equilibrated in 95% solvent A and 5% solvent B. The elution flow rate was 1 mL min^−1^ by linearly increasing of solvent B concentration from 5 to 60% over 17 min, maintained isocratic for 2 min, subsequently increased to 95% over 6 min, then returned to 5% over 3 min and equilibrated for 5 min. The chromatograms were acquired at 280 nm. The instrumentation performance, chromatograms, and initial data processing were carried out with Chromeleon software.

A calibration curve was built using standard solutions of pure oleuropein (2000 ppm), its aglycone form (3,4-DHPEA-EA) (2000 ppm), hydroxytyrosol (2000 ppm), oleacein ((3,4-DHPEA-EDA) (2000 ppm) and demethyloleuropein (2000 ppm) in EtOH; these solutions were then mixed to obtain six standard solutions of 10 ppm, 25 ppm, 50 ppm, 75 ppm, 100 ppm and 125 ppm in both of phenolic compounds. HPLC analysis gave rise to five regression curves (see Supplementary Material). Each DES extract was diluted in 50 μL (in the case of olive leaves) or 500 μL (in the case from ripe drupes) of ethanol, which is a miscible solvent in all the deep eutectic solvents used in this study. Ethanol represents the solvent of choice to avoid the formation of acetals from oleacein; 20 μL of the diluted extracts were analyzed by HPLC and peaks in the chromatograms were identified by comparison with standards.

### 2.6. LC-ESI-QTOF/MS Analysis

Analyses were carried out using an Agilent 6540 UHD Accurate–Mass Q-TOF LC/MS (Agilent, Santa Clara, CA, USA) fitted with an electrospray ionization source (Dual AJS ESI) operating in positive ion mode. Chromatographic separation was achieved using a C_18_ RP analytical column (Poroshell 120, SB-C18, 50 × 2.1 mm, 2.7 μm) at 40 °C with an elution gradient from 20 to 35% of B over 30 min, followed by 35–90% of B over 5 min, with A being H_2_O (0.1% FA) and B CH_3_CN. Flow rate was 0.4 mL/min. The ESI source was operated both in positive ion mode with the following conditions: the fragmentor voltage was set at 120 V, nebulizer gas was set at 35 psig, capillary voltage was set at 3500 V, and drying gas flow rate and temperature were set at 10 dm^3^/min and 300 °C, respectively. For MS/MS measurements collision energy ramp ranging from 15 to 40 eV to promote fragmentation was used. The data were acquired in centroid and profile mode using High Resolution mode (4 GHz). The mass range was set at 50–1000 *m/z* in MS and MS/MS mode. The data were processed with the MassHunter Workstation QualitativeAnalysis.B.03.01 Software. The Q-TOF-MS was calibrated on a daily basis.

### 2.7. Statistical Analysis

The results are expressed by mean ± S.E.M. from at least three independent experiments. For statistical comparisons, quantitative data was analyzed by one-way analysis of variance (ANOVA) followed by Tukey-test according to the statistical program SigmaStat1 (Jandel Scientific, Chicago, IL, USA). A *p*-value less than 0.05 was regarded as significant.

## 3. Results and Discussion

### 3.1. Screening of the NADESs

The structure of NADESs determines their chemical and physical properties, and consequently influences the extraction efficiency of biologically active compounds.

Generally, NADESs are formed of natural, biodegradable and economical compounds. For example, choline chloride (ChCl) is cheap (about 150 €/kg), biodegradable (93% in 14 days), non-toxic (LD50 = 3500 mg/kg) and in addition can be either extracted from biomass or easily obtained from fossil sources. As far as hydrogen bond donors are concerned, the most common ones are urea, carboxylic acids and polyols obtained from renewable sources.

To represent the different “classes” of hydrogen bonds donors typically used in NADES, six different NADESs (Table 1) were chosen based on choline chloride in combination with: urea (NADES-1), glycerol (NADES-2), lactic acid (NADES-3), ethylene glycol (NADES-4), and citric acid (NADES-5).

The NADESs preparation was carried out by simply mixing the two components in a suitable manner and leaving under constant stirring at 80 °C for 2 h until a liquid was formed (Table 1). The prepared NADES were used without the need for purification as extraction solvents for phenolic compounds in olive leaves.

The viscosity of DESs is their main constraint, since it hinders the handling and efficiency as extraction solvents compared to conventional ones. Thus, the addition of water is often performed to adjust the properties of DES and decrease the viscosity and the surface tension. For this reason, a percentage of 20% of water (*w/w*) was set for the screening extraction of the NADESs.

### 3.2. Sample Preparation and Extraction Procedure

Both fresh and properly dried leaves were extracted. The drying process is necessary to remove their water content, with the aim of protecting them from enzymatic degradation. This process was carried out keeping samples in a traditional oven at 80 °C for 45 min.

Then, the leaves were chopped to facilitate the entry of solvents into the cells and thus increase the efficiency of the extraction. The extraction was performed under microwave assistance to favor and accelerate the extraction of phenols present in the plant matrix. In fact, besides the use of green solvents, one of the criteria for the development of an eco-friendly extraction is to reduce energy consumption by using innovative technologies such as microwave-assisted extraction (MAE). MAE has been recognized as an exceptional energy resource for promoting extractions, increasing the yield, the quality of the product and greatly reducing the extraction time.

One of the main factors contributing to the efficiency of microwave extraction is the identification of the optimal extraction temperature [47]. In general, at high temperatures, the solvent power increases because there is a decrease in viscosity and diffusivity, which is very important for viscous solvents such as NADES. An increase in temperature can also cause a reduction in surface tension, as well as a decrease in the interaction between the target compound and the sample matrix, leading to an improvement in the desorption and dissolution of the target compound in the solvent. On the other hand, high temperatures can induce thermal degradation of phenolic compounds. Therefore, we decided to apply, in this study, moderate temperatures equal to 80 °C and the microwave power did not exceed 800 W. The maximum limit of the microwave power used was set in such a way as to avoid overheating of the extraction mixture and the consequent degradation of phenolic compounds.

An extraction time of 30 min is sufficient to obtain maximum yields of phenolic compounds, as well as to obtain a greater extraction of oleuropein in the classic aqueous extraction procedure [12]. Thus, for control, a conventional extraction in water was also performed.

In particular, MAE extraction was carried out by placing 2 g of fresh or dried leaves in a flask equipped with refrigerant in 8 mL of NADES at 80 °C at a power of 800 W. The comparison extraction was carried out by placing, in a flask equipped with coolant, 2 g of fresh or dried leaves in 8 mL of H_2_O at 80 °C at a power of 800 W. The extracts were then centrifuged for 10 min and an aliquot of each extract was finally dissolved in ethanol and directly subjected to HPLC and LC-ESI-QTOF/MS analysis.

### 3.3. HPLC- ESI-QTOF-MS Analysis and Quantitative HPLC

The precise identification of the extracted phenolic compounds is not generally simple as different types of structures are contained. The HPLC-QTOF-MS is a very useful tool in order to characterize natural products. In particular, electrospray ionization (ESI) has been widely applied thanks to its mild ionization technique. An accurate measurement of the mass of small molecules is generally used to determine the elementary formulas, facilitating the identification of unknown substances. The QTOF-MS mass spectrometer combines high sensitivity and mass accuracy for both precursor and fragment ions, providing the elemental composition. This feature helped in the complete identification of the compounds and in the differentiation between the isobaric compounds. The ability of HPLC-ESI-QTOF-MS for qualitative identifications has been reported in several studies [48,49,50].

The UPLC profile of the phenolic compounds of the NADES leaf extracts using MAE are presented in Table 2 where the data obtained from the mass spectra of the identified compounds are summarized, including both the experimental *m/z* values and those calculated for the molecular formulas, and the principal ions, as well as the proposed compound for each peak.

As shown in Table 2, the identified phenolic compounds are almost all derived from Oleuropein.

According to literature data, leaf extracts contain hydroxytyrosol, tyrosol, demethyloleuropein, oleuropein, and one of its isomers, oleacin, oleuropein aglycone (Table 2). It is worth noting that oleuropein is the main phenolic compound identified in all the extracts, and it was identified based on a comparison with the authentic standard. This compound has been described as the major component present in olive leaves. Oleuropein was found in olive leaves together with its isomer.

Another phenolic compound identified in olive tree leaf extracts is tyrosol (NADES-3, NADES-4, NADES-5), which, together with hydroxytyrosol, is in fact one of the best antioxidant phenolic compounds able to prevent natural oxidative processes. Additionally, the presence of the aglycone oleuropein was detected (NADES-2, NADES-3, NADES-4, NADES-5), while demethyloleuropein was detected in green leaves extracted with NADES-1, and in the case of dried leaves when the extraction was performed with NADES-4.

The same extracts were then analyzed by HPLC and the polyphenol content was quantified by comparison with the standards. The results obtained, reported in Table 3, once again show that, as expected, oleuropein is the most abundant component present in olive leaves and that the other components identified with UPLC-MS are instead present in traces.

Generally, the extraction process is greatly affected by the affinity of the DES with the target compounds [51]. In fact, glycerol-based NADES-2 evidenced to be the best solvent for the extraction of oleuropein, followed by NADES-3 and NADES-4. The urea-based NADES and the citric acid-based NADES were discarded due to their low extraction efficiency. The results of the extraction yields are also in agreement with what was predictable based on the viscosity: in fact, the NADES with glycerol, lactic acid are the most efficient and have the lowest viscosity (29.5 and 47.5 cP, respectively), while the other NADES values have higher viscosity values, e.g., NADES with citric acid has viscosity = 448.1 cP) [52]. In the case, instead, of NADES based on urea, the low extraction efficiency is probably due to the lower polarity of NADES compared to those containing polyols or organic acids.

To compare the extraction efficiency of the methodology based on NADES under microwave assistance with the conventional extraction method, a MAE extraction with water was carried out according to protocols already reported in the literature.

**Figure 2:**
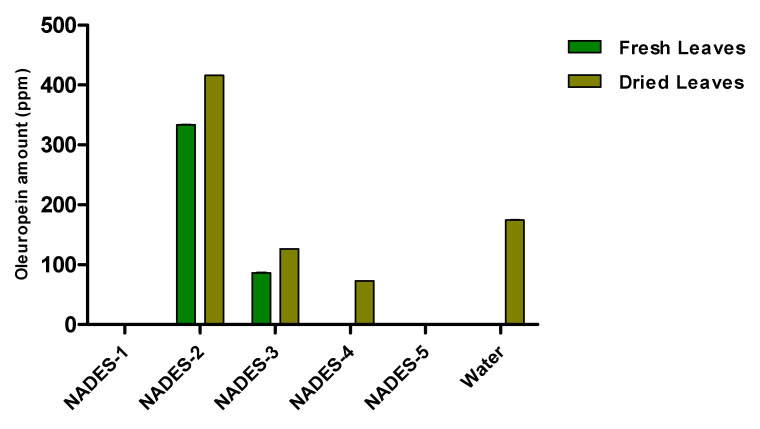
Quantitative analysis of oleuropein in olive leaf extracts carried out by HPLC. Data are expressed as mean ± SD of three independent observations *(p* < 0.05).

The obtained water extract was centrifuged and dried under reduced pressure conditions using a rotary evaporator. The sample was then diluted with the appropriate solvent for the subsequent chromatographic analysis. The phenolic compounds identified and quantified by HPLC in the aqueous extract are oleuropein, as the main component, and traces of hydroxytyrosol and oleacein (Table 3). The construction of a histogram for oleuropein makes it possible to compare the extraction efficiency of the NADESs with that of water (Figure 2).

It is evident from the histogram that NADES-2 consisting of choline chloride and glycerol as a hydrogen bond donor was not only the best among the tested NADESs, but also the green solvent with superior extraction capacity with respect to water (8.31 ± 0.11 ppm versus 5.20 ± 0.10 ppm).

Thus, we decided to apply the same procedure for the extraction of phenolic compounds from ripe olives. The MAE process was performed for 10 and for 30 min in order to validate the procedure, while at the same time verifying the ability of the NADES to convert oleuropein into demethyloleuropein (already present in ripe olives due to the enzymatic activity during ripening), its aglyconic form (3,4-DHPEA-EDA), and hydroxytyrosol. Table 4 reports the phenolic compounds present in the NADES extracts, as well as the phenolics present in water extract.

**Figure 3:**
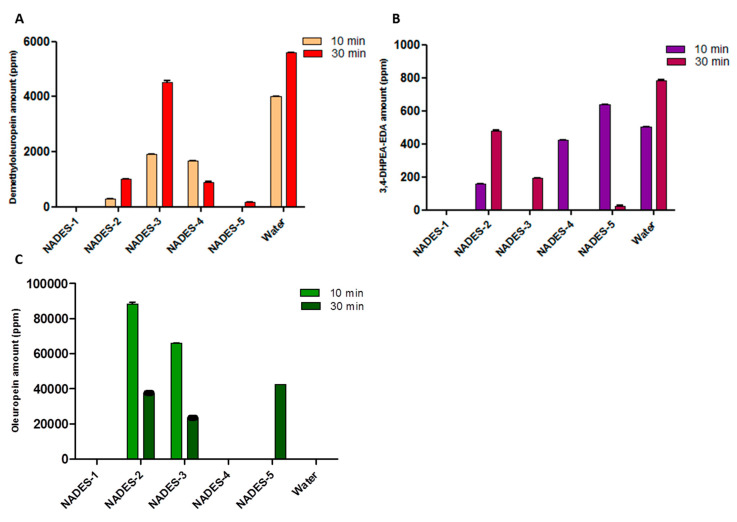
Quantitative analysis, carried out by HPLC, of demethyloleuropein (**A**), 3,4-DHPEA-EDA (**B**) and oleuropein (**C**) in ripe olives extracted with NADESs and water (for control). The extractions were carried out for a duration of 10 and of 30 min.

Even in this case, the construction of histograms for oleuropein, demethyloleuropein and 3,4-DHPEA-EDA amounts allowed us to make a visual comparison between the extraction efficiency of NADESs with that of water (Figure 3).

For all the extracts, the amount of detected hydroxytyrosol is below the limit of detection. Prolonged extraction times resulted in an increase in the amount of phenolic compounds, other than some small discrepancies. When NADES-2 and NADES-3 were used as solvents, an oleuropein-rich extract was obtained after only 10 min. The maximum increase in the yield of oleuropein was surprising in the case of NADES-2 (88287.57 ± 0.24 ppm of oleuropein) as it was a yield that practically doubled that obtained with a conventional water extraction carried out for 30 min.

In addition, a decrease of oleuropein content over time (after 30 min) was observed with a concomitant increase of the amount of demethyloleuropein. Excellent results were obtained in the extraction process of demethyloleuropein using NADES-3 after 30 min of extraction.

In the final part of our study, we performed some experiments to assess the extraction efficiency of NADES with the addition of a certain quantity of water. The high viscosity of NADES would limit the transfer of the target compound from the natural matrix.

The water addition is a pivotal factor to reduce the DES’s viscosity since the interactions (e.g., hydrogen bonding) between the components of the DES are weaken in presence of water. To tackle this issue, we attempted the extraction using aqueous solutions of NADES. According to the literature, a percentage of 20% water content [53] can sufficiently reduce the viscosity of the NADES and meanwhile maintain the hydrogen-bonding network. Higher amount of water could gradually weaken the interactions between the components of the DESs.

This test was carried out by extracting the ripe olive samples with the NADES added with 20% (*w/w*) of water. All of the tested NADES, except for NADES-1, were only able to extract demethyloleuropein and 3,4-DHPEA-EDA (Table 5).

Actually, quantitative analyses on all the extracts obtained from the new NADESs do not reveal the presence of oleuropein and, anyhow, the quantity of demethyloleuropein determined is lower than that determined in the classic aqueous extract. However, the extracts obtained using NADES-2-W and NADES-3-W show the presence of 3,4-DHPEA-EDA, which is not identified in the classic aqueous extraction (water). Furthermore, once again NADES-2-W, which has glycerol as its component, shows that after 30 min of extraction, the quantity of demethyloleuropein decreases, while the quantity of 3,4-DHPEA-EDA increases (compared to the quantities detected after 10 min extraction).

This result leads us to believe that NADES-2-W, in addition to the function of extraction solvent, favors the formation of oleacein (3,4-DHPEA-EDA) from demethyloleuropein through the synthetic transformation already reported [18].

## 4. Conclusions

The results presented in this work proved that the combination of sustainable green solvents (NADESs) and microwave assisted extraction techniques (MAE) is an efficient approach for recovering phenolic compounds from olive oil processing wastes.

Phenolic compounds are polar molecules and the differences observed in the extraction efficiency of various NADESs could be explained because their chemical and physical properties are determined by their chemical form. In general, the functional groups involved in the hydrogen bonds are the hydroxyl, amidic and carboxylic groups which are abundant in NADES and are obviously present in phenolic compounds. H-bonding interactions between molecules of NADES and phenolic compounds are responsible for their extractability.

The urea-based NADES-1 was discarded due to its poor power of extraction. The polyols-based NADESs, NADES-2 and NADES-4, as well as organic acid-based NADES-3, proved to be effective in the extraction of phenolic compounds from olive leaves and drupes. Glycerol-based NADES-2 was confirmed to be an excellent solvent, and was even more effective than conventional solvent.

The proposed NADES-based microwave-assisted extraction process is highly efficient and really eco-friendly. The natural origin of the components of the NADES makes them attractive for further applications in cosmetics, pharmaceuticals and food industries. The NADES extract could be used in their wholeness, even in products to be used or consumed by people.

## Figures and Tables

**Table 1:** Compositions and abbreviations for the prepared NADESs.

Composition HBA: HBD	Molar Ratio	Water Addition (%)	Acronym	Physical Aspect Color
ChCl: Urea	1:2	0	NADES-1	Colorless transparent oil
ChCl: Urea	1:2	20	NADES-1-W	Colorless transparent oil
ChCl: Glycerol	1:1	0	NADES-2	Colorless transparent oil
ChCl: Glycerol	1:1	20	NADES-2-W	Colorless transparent oil
ChCl: Lactic acid	1:1	0	NADES-3	Colorless transparent oil
ChCl: Lactic acid	1:1	20	NADES-3-W	Colorless transparent oil
ChCl: Ethylene glycol	1:1	0	NADES-4	Colorless transparent oil
ChCl: Ethylene glycol	1:1	20	NADES-4-W	Colorless transparent oil
ChCl: Citric acid	1:1	0	NADES-5	Pale yellow semisolid
ChCl: Citric acid	1:1	20	NADES-5-W	Pale yellow oil

**Table 2:** Compounds identified by UPLC- ESI-QTOF-MS analysis in olive leaf extracts extracted with NADESs.

Compound	Htytrosol	Tyrosol	Demethyloleuropein	Oleuropein	3,4-DHPEA-EDA	Isomer Oleuropein	3,4-DHPEA-EA
	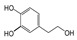	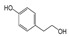	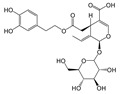	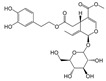	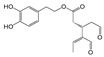	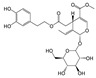	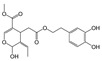
**r.t. *min***	2.47	2.95	3.36	3.89	3.97	4.03	5.10
**Formula**	C_8_H_10_O_3_	C_8_H_10_O_2_	C_24_H_30_O_13_	C_25_H_32_O_13_	C_17_H_20_O_6_	C_25_H_32_O_13_	C_19_H_22_O_8_
**Theorical** ***m/z***	154.0630	138.0681	526.1686	540.1843	320.1260	540.1843	378.1315
**Measured** ***m/z***	154.0634	138.0673	526.1670	540.1828	320.1253	540.1833	378.1309
**MS spectrum peak list**	177.0511 (M+Na)^+^	139.0744 (M+H)^+^ 161.0576 (M+Na)^+^	527.1760 (M+H)^+^ 549.1557 (M+Na)^+^	563.1726 (M+Na)^+^	343.1138 (M+H)^+^	563.1727 (M+Na)^+^	401.1200 (M+Na)^+^
**Fresh Leaves**	NADES-4, NADES-5	NADES-3, NADES-4, NADES-5	NADES-1	NADES-1, NADES-2, NADES-3, NADES-4	NADES-3, NADES-4, NADES-5	NADES-1, NADES-2, NADES-3, NADES-4	NADES-2, NADES-3, NADES-4, NADES-5
**Dried Leaves**	NADES-2, NADES-4, NADES-5	NADES-2, NADES-3	NADES-4	NADES-2, NADES-3, NADES-4		NADES-2, NADES-3, NADES-4	NADES-2

**Table 3:** Content of phenolic compounds in the examined olive leaf extracts (in NADESs or water) from HPLC analysis ^a^.

Solvent		Oleuropein [ppm] ^b^
**NADES-1**	Fresh Leaves	ND
	Dried Leaves	ND
**NADES-2**	Fresh Leaves	333.73 ± 0.17
	Dried Leaves	416.08 ± 0.15
**NADES-3**	Fresh Leaves	86.30 ± 1.12
	Dried Leaves	126.51 ± 0.10
**NADES-4**	Fresh Leaves	ND
	Dried Leaves	72.97 ± 0.05
**NADES-5**	Fresh Leaves	ND
	Dried Leaves	ND
**Water**	Fresh Leaves	ND
	Dried Leaves	174.47 ± 0.42

^a^ Htyrosol, tyrosol, demethyloleuropein, 3,4-DHPEA-EA, 3,4-DHPEA-EDA are in an amount not detectable by HPLC. ^b^ Data are expressed as the means ± SD of three independent observations. (*p* < 0.05). ND = not detected.

**Table 4:** Content of phenolic compounds in the examined ripe olive extracts (in NADESs or water) from HPLC analysis ^a^.

Solvent	Time (min)	Demethyloleuropein [ppm] ^b^	3,4-DHPEA-EDA [ppm] ^b^	Oleuropein [ppm] ^b^
**NADES-1**	10	ND	ND	ND
	30	ND	ND	ND
**NADES-2**	10	284.67 ± 1.13	156.64 ± 1.81	88320.90 ± 38.03
	30	1019.84 ± 0.53	480.60 ± 0.55	37602.42 ± 1.21
**NADES-3**	10	1918.761 ± 1.88	ND	52643.39 ± 51.7
	30	4527.07 ± 18.19	193.15 ± 0.55	23338.72 ± 10.50
**NADES-4**	10	1655.97 ± 2.03	420.92 ± 1.92	ND
	30	903.79 ± 2.15	ND	ND
**NADES-5**	10	ND	637.06 ± 1.74	ND
	30	174.95 ± 0.93	25.69 ± 1.73	42534.86 ± 12.12
**Water**	10	4000.52 ± 2.15	503.05 ± 1.31	ND
	30	5602.64 ± 8.72	783.25 ± 3.23	ND

^a^ Htyrosol, tyrosol, 3,4-DHPEA-EDA are in an amount not detectable by HPLC. ^b^ Data are expressed as mean ± SD based on triplicate values (*p* < 0.05). ND = Not Detected.

**Table 5:** Content of phenolic compounds extracted in the ripe olives (in NADES added with 20% water) from HPLC analysis ^a^.

Solvent	Time min.	Demethyloleuropein [ppm] ^b^	3,4-DHPEA-EDA [ppm] ^b^
**NADES-1-W**	10	ND	ND
	30	ND	ND
**NADES-2-W**	10	1309.518 ± 2.124	198.3333 ± 0.994
	30	869.8605 ± 3.384	254.4684 ± 1.384
**NADES-3-W**	10	567.562 ± 1.158	469.912 ± 2.143
	30	338.0844 ± 2.964	438.5344 ± 1.951
**NADES-4-W**	10	303.2958 ± 3.121	154.5307 ± 1.005
	30	241.5935 ± 4.001	105.9052± 1.805
**NADES-5-W**	10	1303.292 ± 2.021	ND
	30	2125.9 ± 1.976	ND

^a^ Htyrosol, Oleuropein, 3,4-DHPEA-EA are in an amount not detectable by HPLC. ^b^ Data are expressed as mean ± SD of three independent observations (*p* < 0.05). ND = Not Detected.

## Supplementary Materials

The following are available online at https://www.mdpi.com/2076-3921/9/6/513/s1.
