# Supplementary material for: Natural Deep Eutectic Solvent as Extraction Media for the Main Phenolic Compounds from Olive Oil Processing Wastes
Source: Antioxidants (Basel). 2020 Jun 11;9(6):513. doi: 10.3390/antiox9060513 (PMC7346206; doi:10.3390/antiox9060513)
Supplement: Supplementary file 1 [file antioxidants-09-00513-s001.pdf]

Supplementary Materials

Natural Deep Eutectic Solvent as Extraction Media for the Main Phenolic Compounds from Olive Oil Processing Wastes.

Sonia Bonacci [1], Maria Luisa Di Gioia [2]*, Paola Costanzo [1]*, Loredana Maiuolo [3], Sofia Tallarico [1] and Monica Nardi [1]

[1] Dipartimento di Scienze della Salute, Università Magna Græcia, Viale Europa, 88100-Germaneto (CZ), Italy; s.bonacci@unicz.it (S.B.); pcostanzo@unicz.it (P.C.); monica.nardi@unicz.it (M.N.); s.talalrico@unicz.it (S.T.)

[2] Dipartimento di Farmacia e Scienze della Salute e della Nutrizione, Edificio Polifunzionale, Università della Calabria, 87030 Arcavacata di Rende, Cosenza.; ml.digioia@unical.it (M.L.D.G.)

[3] Dipartimento di Chimica e Tecnologie Chimiche, Università della Calabria, Cubo 12C, 87036-Arcavacata di Rende (CS), Italy; loredana.maiuolo@unical.it (L.M.)

* Correspondence: ml.digioia@unical.it; Tel.: +39 0984 493095 (M.L.D.G.); pcostanzo@unicz.it; Tel.: +39 0961 3694094 (P.C.).

LC-ESI-QTOF/MS analysis

Compound Label	Name	*m/z*	RT	Algorithm	Mass
hydroxytyrosol	**hydroxytyrosol**	177,0511	2,47	Find By Formula	154,06

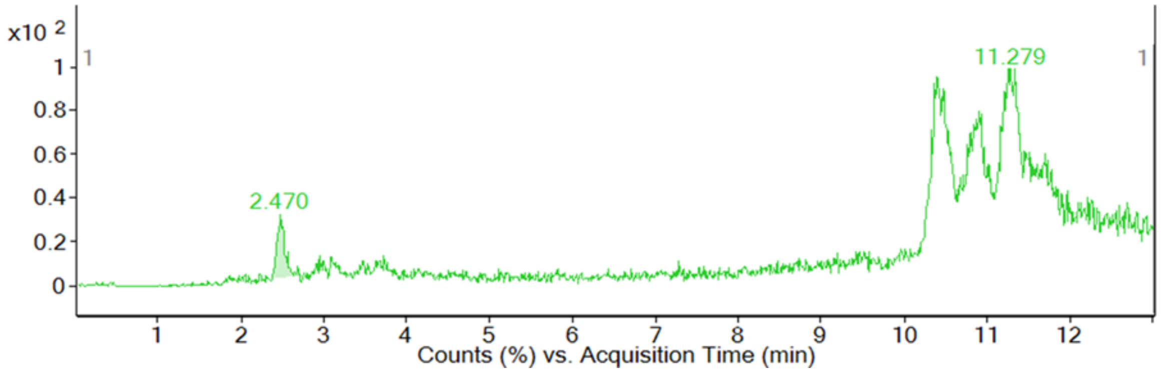

MS Zoomed Spectrum

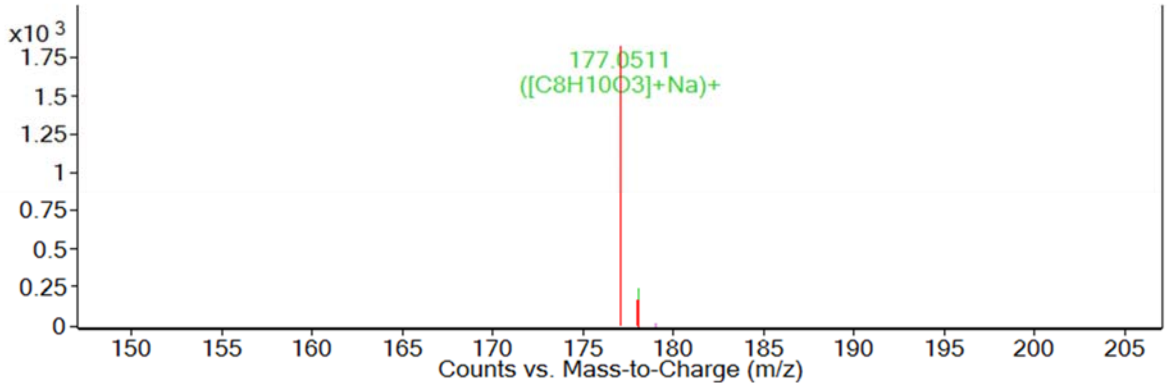

MS Spectrum Peak List

m/z	z	Abund	Formula	Ion
177,0511	1	1826,29	C8H10O3	(M+Na)+
178,0547	1	252,82	C8H10O3	(M+Na)+

Compound Label	Name	*m/z*	RT	Algorithm	Mass
tyrosol	**tyrosol**	139,0745	2,899	Find By Formula	138,0672

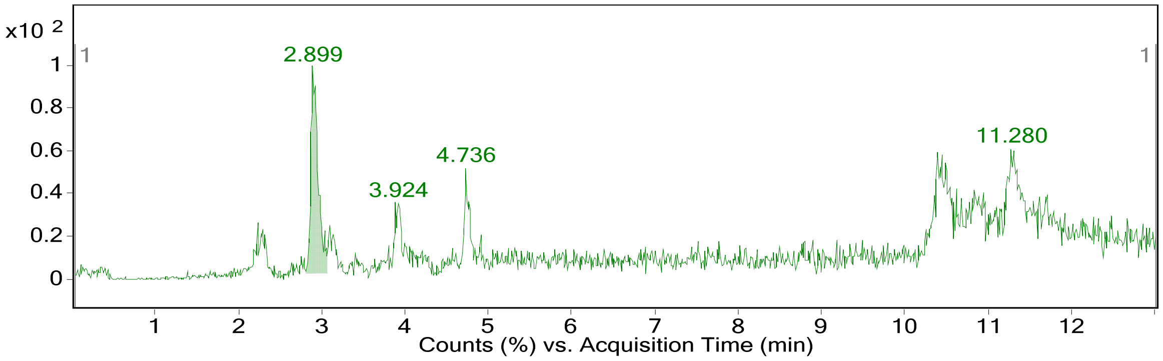

MS Zoomed Spectrum

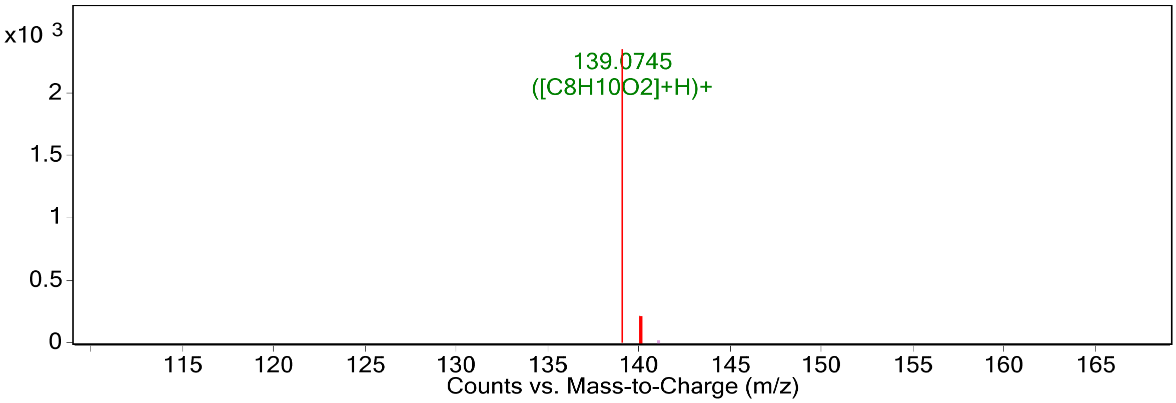

MS Spectrum Peak List

m/z	z	Abund	Formula	Ion
139,0745	1	2344,66	C8H10O2	(M+H)+
140,0783	1	214,07	C8H10O2	(M+H)+

Compound Label	Name	*m/z*	RT	Algorithm	Mass
demethyloleuropein	**demethyloleuropein**	549,1557	3,362	Find By Formula	526,167

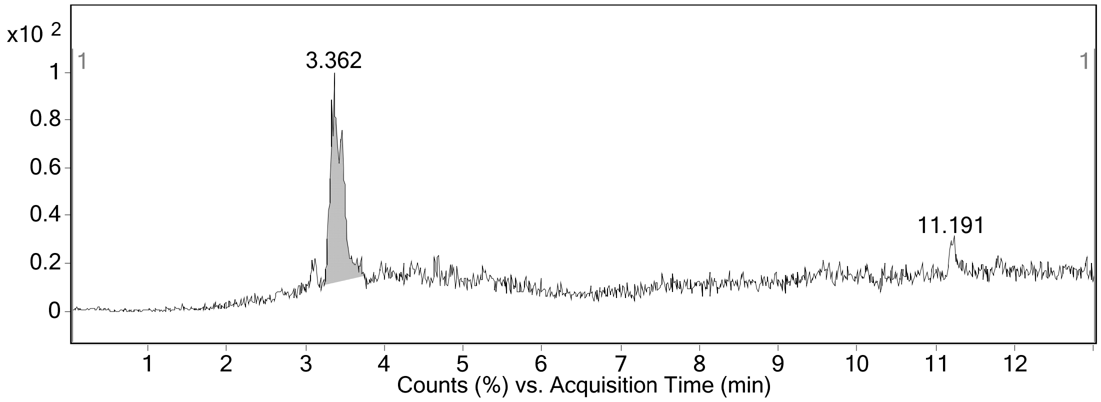

MS Spectrum

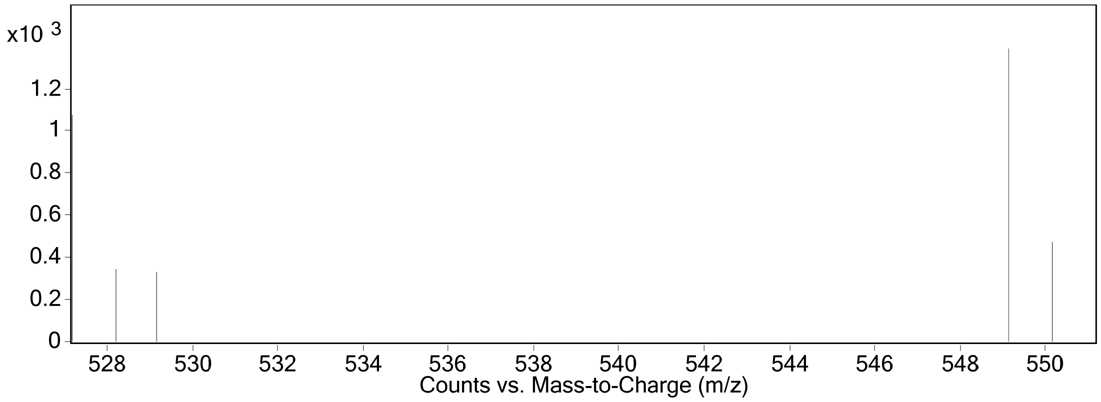

MS Zoomed Spectrum

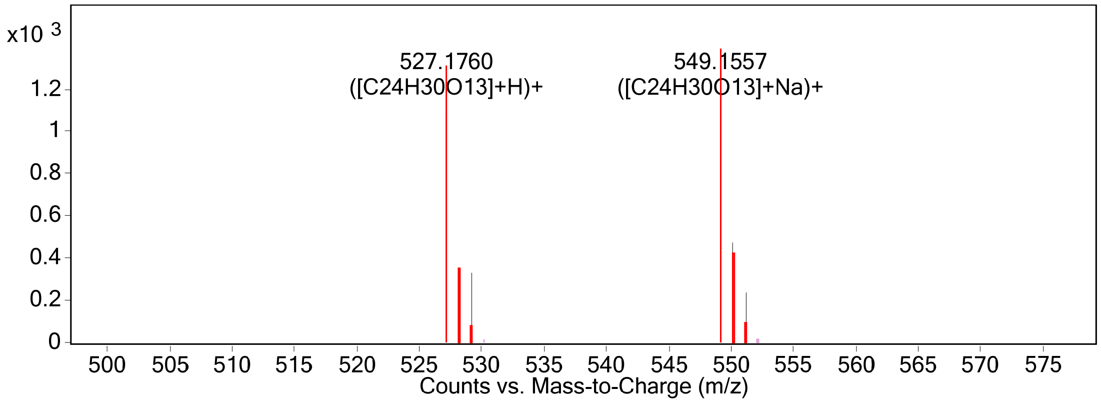

MS Spectrum Peak List

m/z	z	Abund	Formula	Ion
527,176	1	1078,15	C24H30O13	(M+H)+
528,1856	1	341,51	C24H30O13	(M+H)+
529,1651	1	326,37	C24H30O13	(M+H)+
549,1557	1	1389,97	C24H30O13	(M+Na)+
550,1609	1	471,72	C24H30O13	(M+Na)+
551,1642	1	233,13	C24H30O13	(M+Na)+

Compound Label	Name	*m/z*	RT	Algorithm	Mass

oleuropein	**oleuropein**	563,1735	3,856	Find By Formula	540,1839

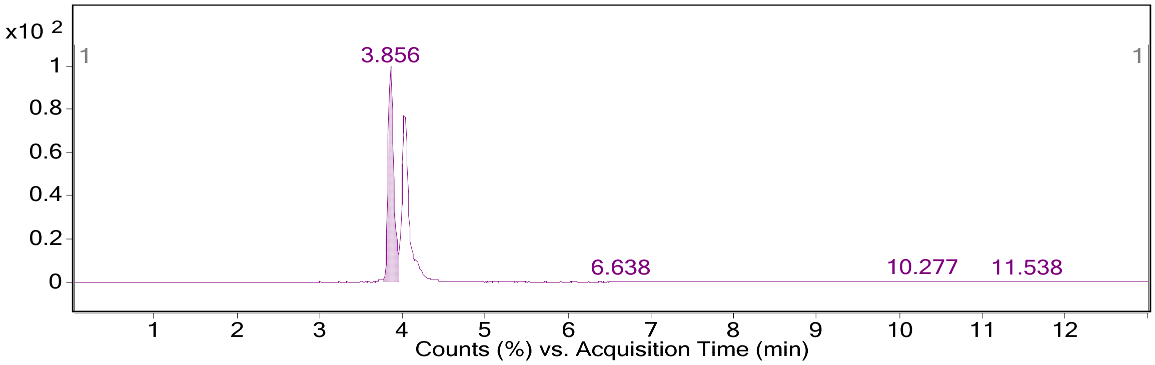

MS
Spectrum

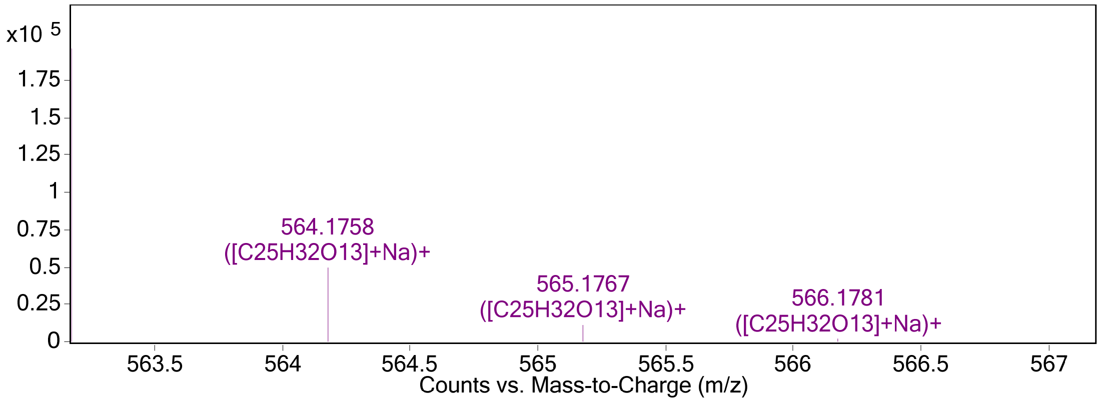

MS Zoomed Spectrum

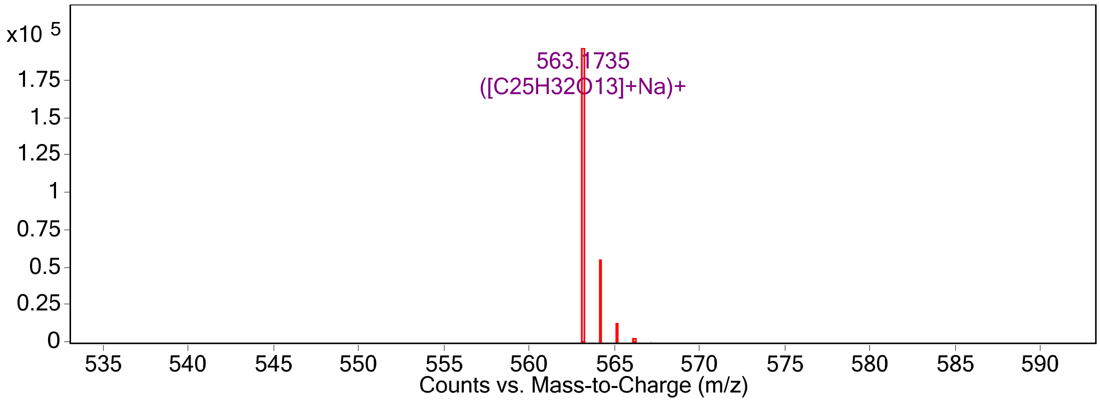

MS Spectrum Peak List

m/z	**z**	**Abund**	**Formula**	**Ion**
563,1735	1	196279,95	C25H32O13	(M+Na)+
564,1758	1	49767,15	C25H32O13	(M+Na)+
565,1767	1	11598,06	C25H32O13	(M+Na)+
566,1781	1	1971,54	C25H32O13	(M+Na)+
567,18	1	324,16	C25H32O13	(M+Na)+

Compound Label	Name	*m/z*	RT	Algorithm	Mass		
oleacein	**oleacein**	343,1136	3,957	Find By Formula	320,1254		

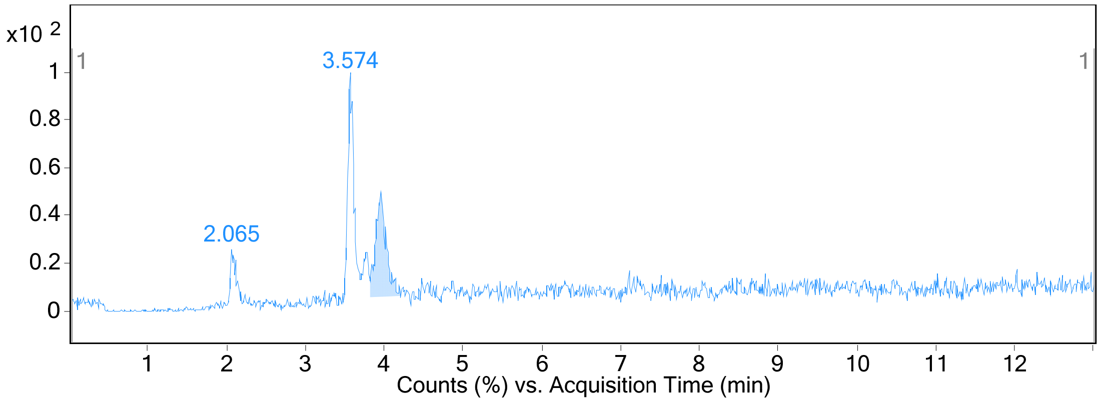

MS Zoomed Spectrum

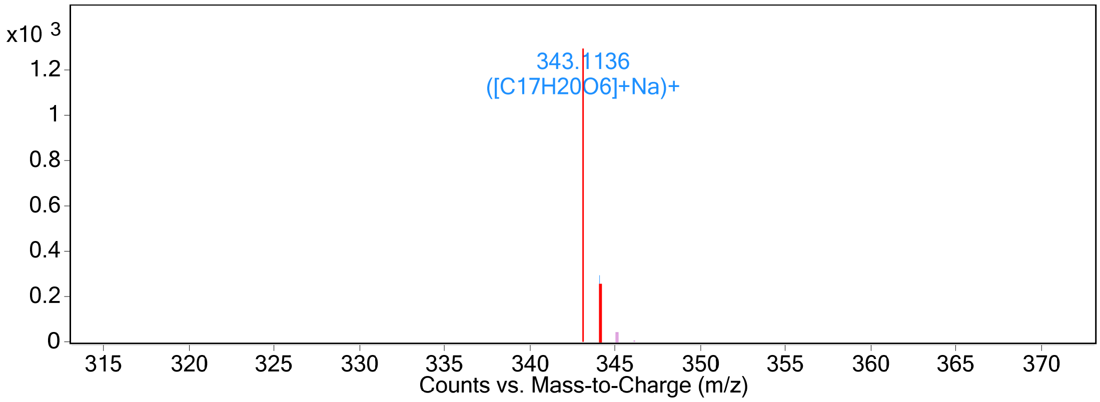

MS Spectrum Peak List

m/z	z	Abund	Formula	Ion
343,1136	1	1292,55	C17H20O6	(M+Na)+
344,1225	1	293,47	C17H20O6	(M+Na)+

**Compound Label**	**Name**	***m/z***	**RT**	**Algorithm**	**Mass**
oleuropein isomer	**Oleuropein**	563,1727	4,025	Find By Formula	540,183

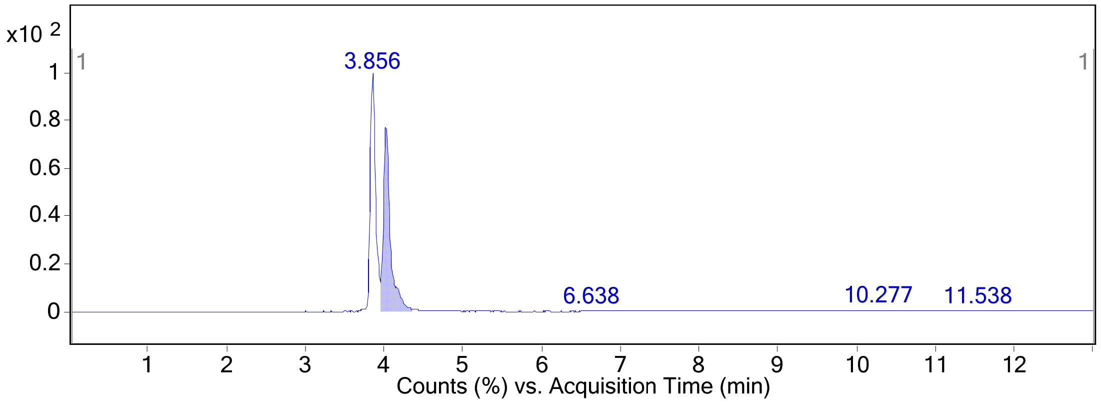

MS Spectrum

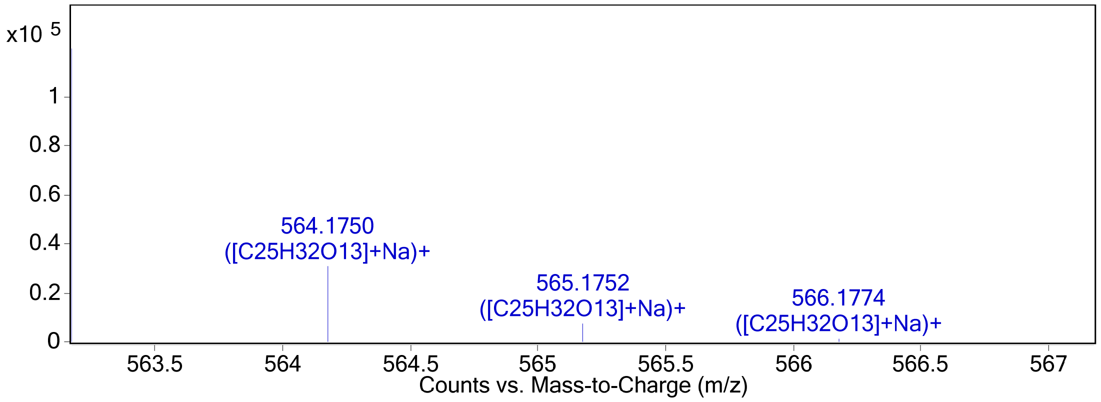

MS Zoomed Spectrum

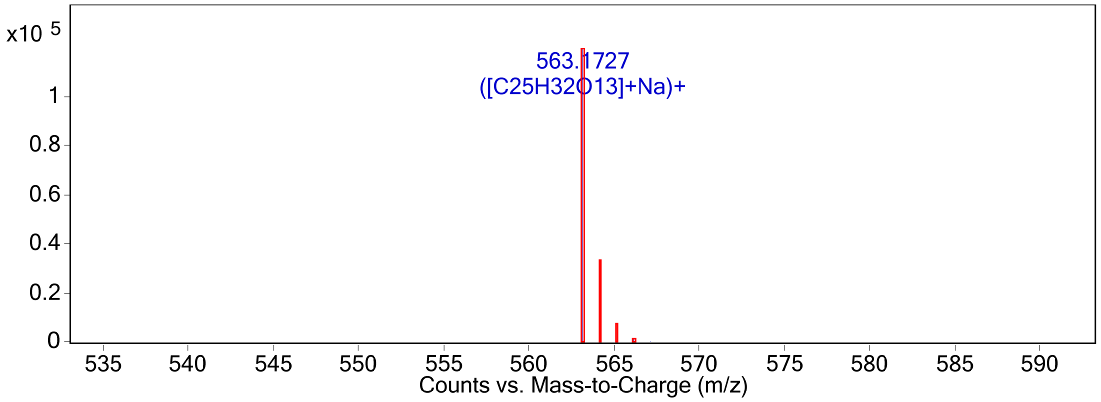

MS Spectrum Peak List

m/z	z	Abund	Formula	Ion
563,1727	1	119782,23	C25H32O13	(M+Na)+
564,175	1	30758,26	C25H32O13	(M+Na)+
565,1752	1	7501,86	C25H32O13	(M+Na)+
566,1774	1	1304,45	C25H32O13	(M+Na)+
567,1769	1	289,93	C25H32O13	(M+Na)+

Compound Label	Name	*m/z*	RT	Algorithm	Mass
Oleuropein Aglycone	**Oleuropein Aglycone**	401,1172	5,107	Find By Formula	378,1278

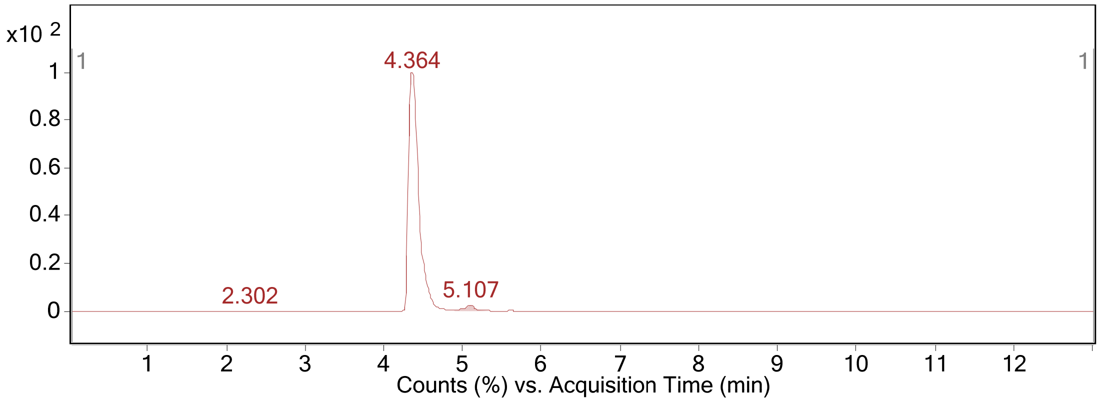

MS Spectrum

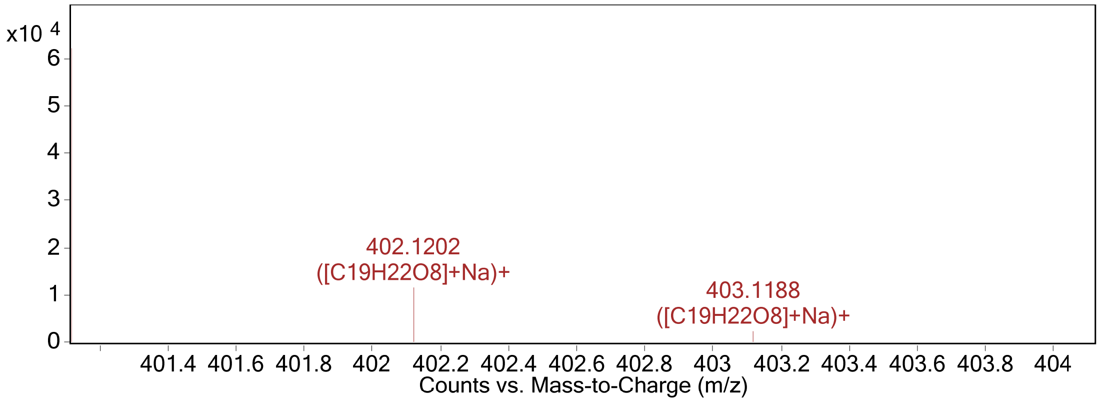

MS Zoomed Spectrum

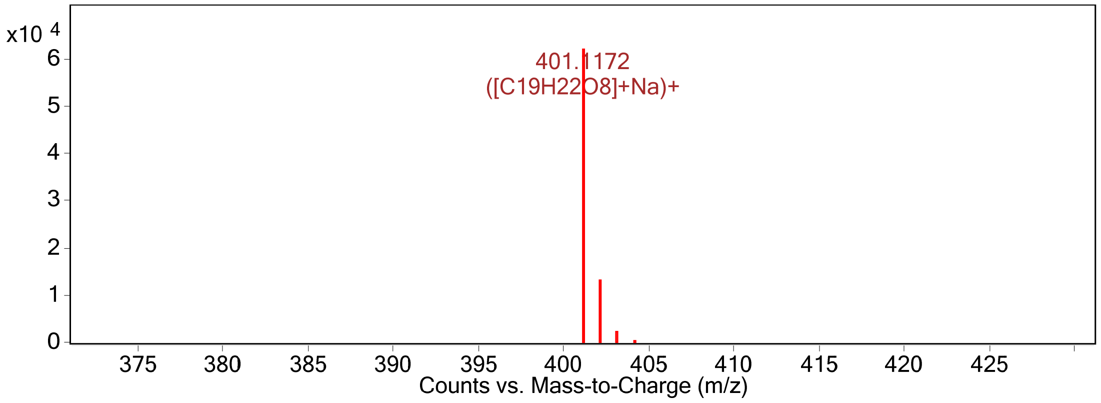

MS Spectrum Peak List

m/z	z	Abund	Formula	Ion
401,1172	1	62217,47	C19H22O8	(M+Na)+
402,1202	1	11596,97	C19H22O8	(M+Na)+
403,1188	1	2325,22	C19H22O8	(M+Na)+
404,1184	1	321,16	C19H22O8	(M+Na)+

HPLC analysis

HPLC analysis was performed using Thermo Scientific (Rodano, MI, Italy) Dionex Ultimate 3000, equipped with a 25 cm×4.6 mm Thermo Scientific Hypersil GOLD C18 column packed with 5 μm particles. For HPLC separation of of the phenolic compounds in DESs, a gradient elution with a mixture of solvents A (H_2O/trifluoroacetic acid, pH=2,46) and B (acetonitrile) was used. The column was equilibrated in 95% solvent A and 5% solvent B. The elution flow rate was 1 ml min^{-1} by linearly increasing of solvent B concentration from 5 to 60% in 17 min, remain in isocratic for 2 min, subsequently increase to 95% in 6 minutes, then return at 5% in 3 min and equilibrated in 5 min. The chromatograms were acquired at 280nm. The instrumentation performance, chromatograms, and initial data processing were carried out with Chromeleon software.

A calibration curve was built using standard solutions of pure oleuropein (2000 ppm), its aglycone (3,4-DHPEA-EA) (2000 ppm), hydroxytyrosol (2000 ppm), oleacein ((3,4-DHPEA-EDA) (2000 ppm) and demethyloleuropein (2000 ppm) in EtOH; these solutions were then mixed to obtain six standard solutions of 10 ppm, 25 ppm, 50 ppm,75 ppm, 100 ppm and 125 ppm in both of phenolic compounds. HPLC analysis gave rise to five regression curves (Fig. 1Sa, 1Sb, 1Sc, 1Sd, 1Se).

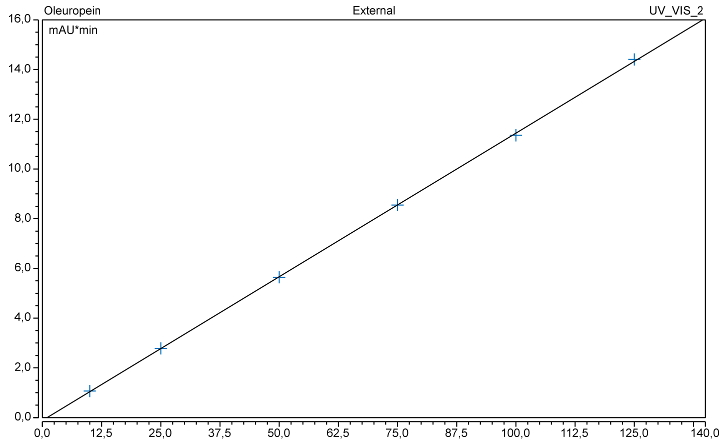

Figure 1Sa: Linear plot of oleuropein concentration vs area. R^2=0,99989.

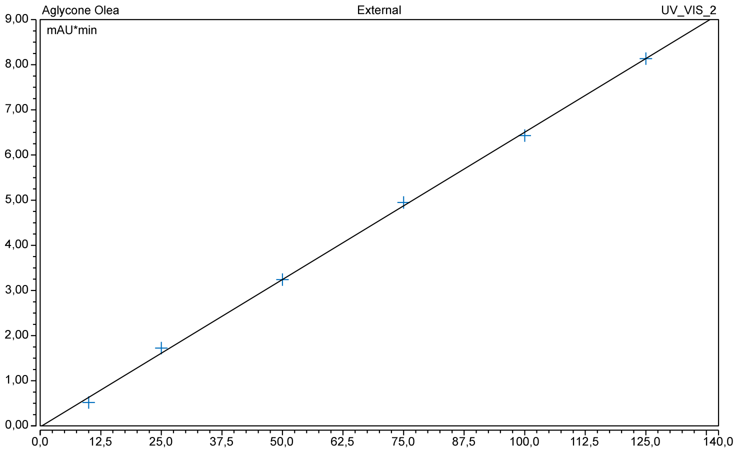

Figure 1Sb: Linear plot of oleuropein agl. (3,4-DHPEA-EA) concentration vs area. R^2=0,99911.

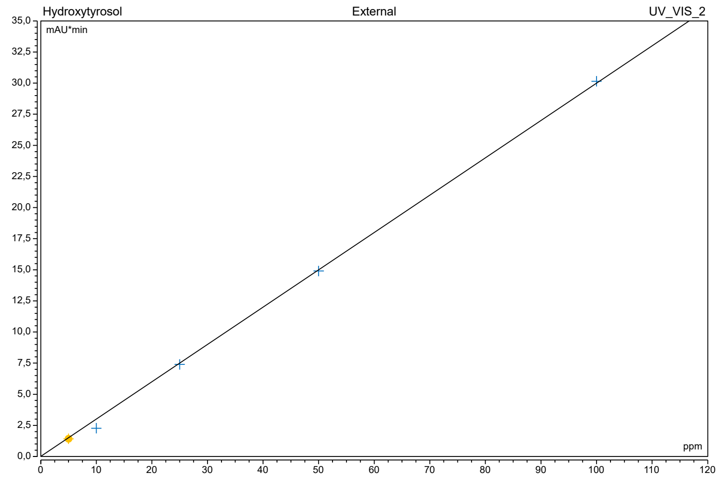

Figure 1Sc: Linear plot of Hydroxytyrosol concentration vs area. R^2=0,99897

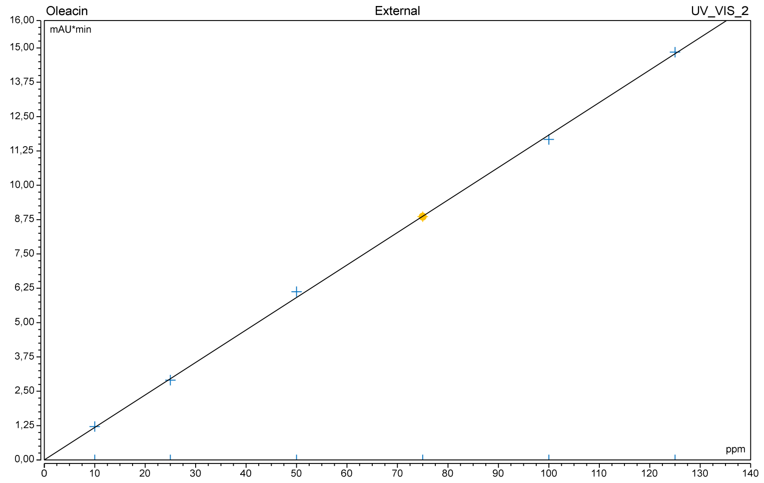

Figure 1Sd: Linear plot of oleacein (3,4-DHPEA-EDA) concentration vs area. R^2=0,99943.

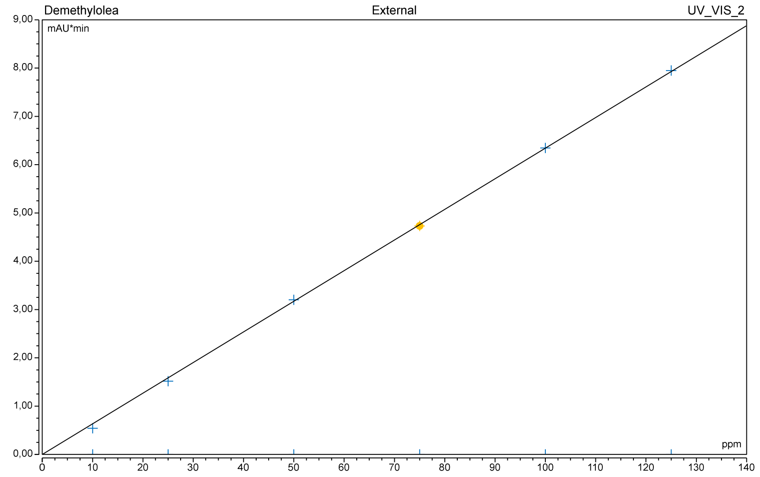

Figure 1Se: Linear plot of demethyloleuropein concentration vs area. R^2=0,99962

The crude of reaction carried out for each DES was diluted in 50 μl of ethanol, which is a miscible solvent in all the deep eutectic solvents used in this study, and also represent the best choice in order to preserve the oleacein from the acetal formation; subsequently 20 μl were analyzed by HPLC. The chromatographic profile of the reaction mixture was characterized by comparison to those with standard compounds.

NADES-1

Dried Leaves

Chromatogram and Results			
**Injection Details**			
Injection Name:	ChCL+Urea+ (F.S.)_in 50 uL_04-12-18	*Run Time (min):*	35,00
Vial Number:	27	*Injection Volume:*	20,00
Injection Type:	Unknown	*Channel:*	UV_VIS_2
Calibration Level:		*Wavelength:*	280
Instrument Method:	Oleaolna MIX OK	*Bandwidth:*	2
Processing Method:	Prova	*Dilution Factor:*	1,0000
Injection Date/Time:	04/dic/18 13:17	*Sample Weight:*	1,0000

Chromatogram

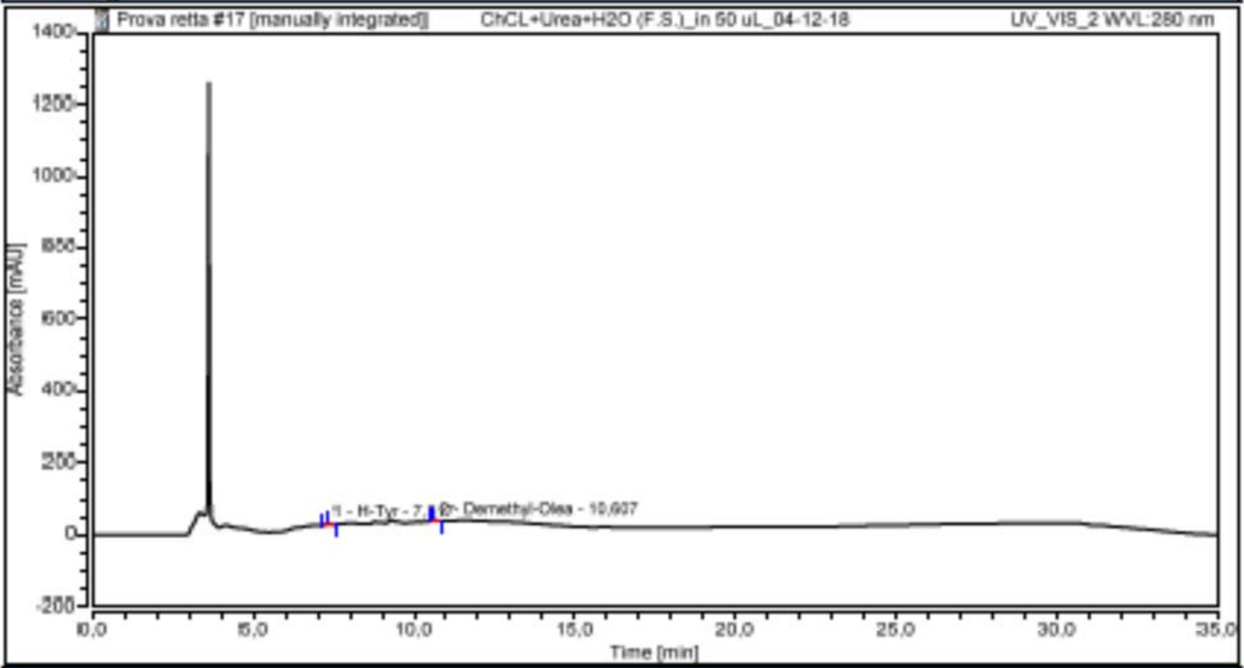

Integration Results

No.	Peak Name	Retention Time min	Area mAU*min	Height mAU	Relative Area %	Relative Height %	Amount
1	H-Tyr	7,317	1,061	5,596	58,36	56,38	n.a.
n.a.	Tyrosol	n.a.	n.a.	n.a.	n.a.	n.a.	n.a.
2	Demethyl-Olea	10,607	0,757	4,329	41,64	43,62	n.a.
n.a.	Olea	n.a.	n.a.	n.a.	n.a.	n.a.	n.a.
n.a.	Oleacein	n.a.	n.a.	n.a.	n.a.	n.a.	n.a.
n.a.	Aglicone Olea	n.a.	n.a.	n.a.	n.a.	n.a.	n.a.
Total:			1,818	9,926	100,00	100,00	

NADES-1

Fresh Leaves

Chromatogram and Results

Injection Details

Injection Name:	**ChCL+Urea 10 min(F.V.)_in 50 uL_04-12-18**	*Run Time (min):*	**35,00**
Vial Number:	**29**	*Injection Volume:*	**20,00**
Injection Type:	**Unknown**	*Channel:*	**UV_VIS_2**
Calibration Level:		*Wavelength:*	**280**
Instrument Method:	**Oleaoina MIX OK**	*Bandwidth:*	**2**
Processing Method:	**Prova**	*Dilution Factor:*	**1,0000**
Injection Date/Time:	**04/dic/18 16:14**	*Sample Weight:*	**1,0000**

Chromatogram

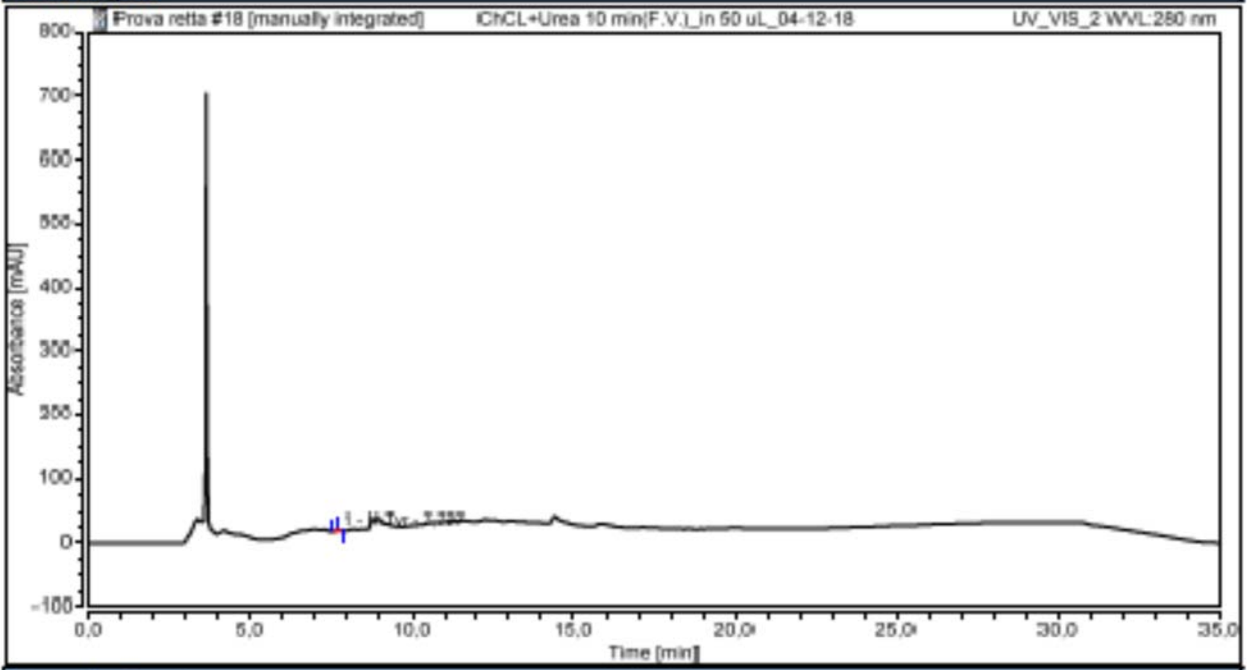

Integration Results

No.	Peak Name	Retention Time min	Area mAU*min	Height mAU	Relative Area %	Relative Height %	Amount
1	H-Tyr	7,727	0,212	1,370	100,00	100,00	n.a.
n.a.	Tyrosol	n.a.	n.a.	n.a.	n.a.	n.a.	n.a.
n.a.	Demethyl-Olea	n.a.	n.a.	n.a.	n.a.	n.a.	n.a.
n.a.	Olea	n.a.	n.a.	n.a.	n.a.	n.a.	n.a.
n.a.	Oleacein	n.a.	n.a.	n.a.	n.a.	n.a.	n.a.
n.a.	Aglicone Olea	n.a.	n.a.	n.a.	n.a.	n.a.	n.a.
**Total:**			**0,212**	**1,370**	**100,00**	**100,00**	

NADES-2

Dried Leaves

Chromatogram and Results

Injection Details

Injection Name:	ChCl+Glicerolo (F.S.)_In 60 uL_27-11-18	*Run Time (min):*	36,00
Vial Number:	22	*Injection Volume:*	20,00
Injection Type:	Unknown	*Channel:*	UV_VIS_2
Calibration Level:		*Wavelength:*	280
Instrument Method:	Oleaolna MIX OK	*Bandwidth:*	2
Processing Method:	Prova	*Dilution Factor:*	1,0000
Injection Date/Time:	27/nov/18 14:24	*Sample Weight:*	1,0000

Chromatogram

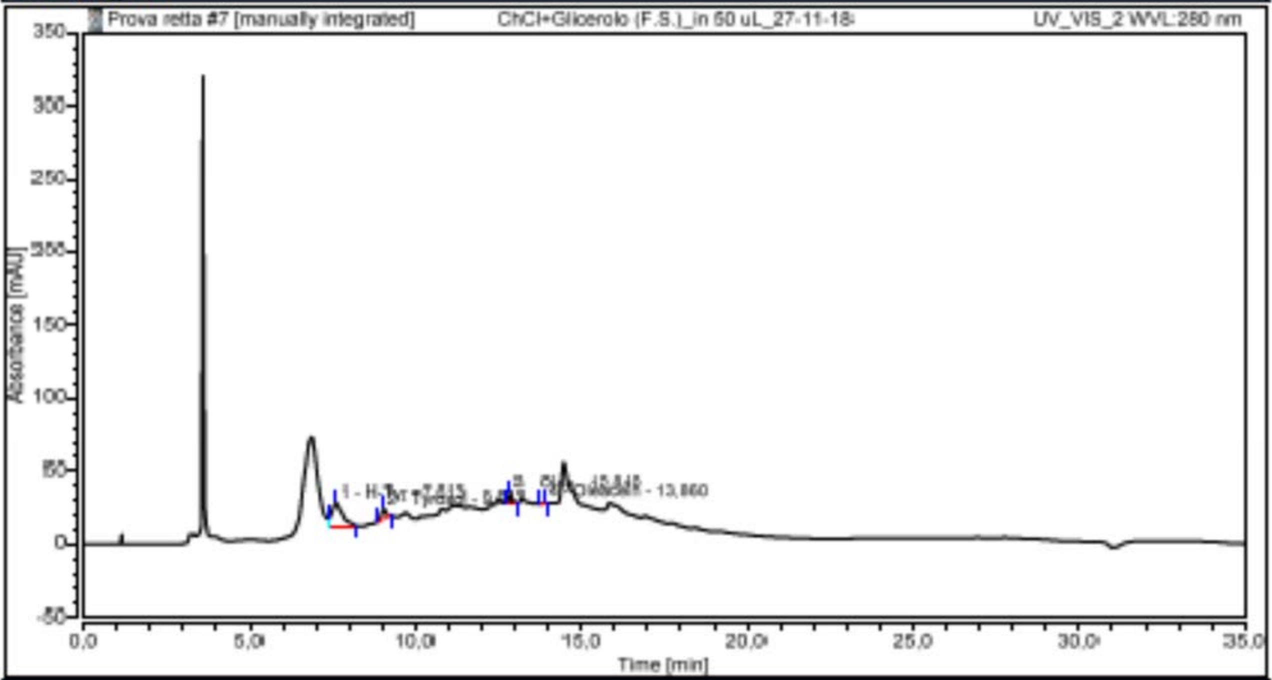

Integration Results

No.	Peak Name	Retention Time min	Area mAU*min	Height mAU	Relative Area %	Relative Height %	Amount
1	H-Tyr	7,613	5,959	15,784	74,51	53,59	n.a.
2	Tyrosol	9,047	1,036	6,563	12,95	22,28	n.a.
n.a.	Demethyl-Olea	n.a.	n.a.	n.a.	n.a.	n.a.	n.a.
3	Olea	12,840	0,846	6,132	10,58	20,82	8,3165
4	Oleacein	13,860	0,157	0,972	1,96	3,30	n.a.
n.a.	Aglicone Olea	n.a.	n.a.	n.a.	n.a.	n.a.	n.a.
Total:			7,998	29,451	100,00	100,00	

NADES-2

Fresh leaves

Chromatogram and Results

Injection Details

Injection Name:	ChCl+Glicerolo (F.V.)_in 50uL_27-11-18	*Run Time (min):*	35,00
Vial Number:	22	*Injection Volume:*	20,00
Injection Type:	Unknown	*Channel:*	UV_VIS_2
Calibration Level:		*Wavelength:*	280
Instrument Method:	Oleaolna MIX OK	*Bandwidth:*	2
Processing Method:	Prova	*Dilution Factor:*	1,0000
Injection Date/Time:	27/nov/18 15:35	*Sample Weight:*	1,0000

Chromatogram

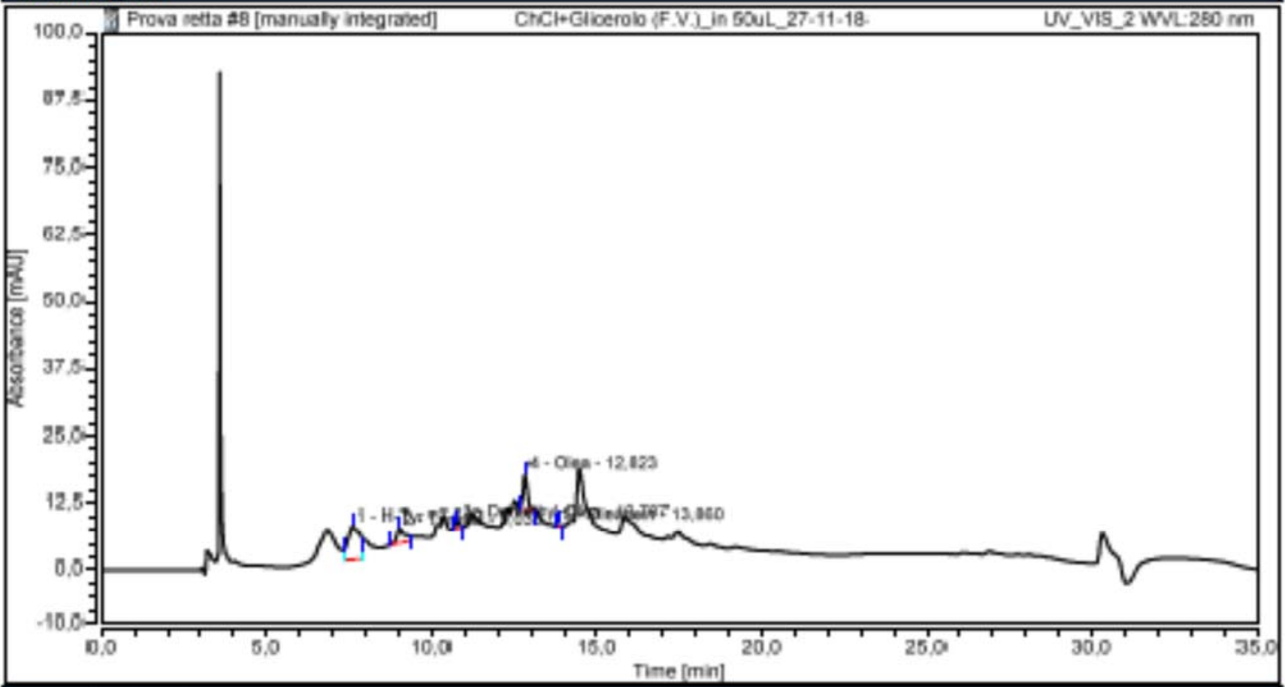

Integration Results

No.	Peak Name	Retention Time<br>min	Area<br>mAU*min	Height<br>mAU	Relative Area<br>%	Relative Height<br>%	Amount
1	H-Tyr	7,610	2,721	6,253	57,62	36,90	n.a.
2	Tyrosol	9,033	0,756	2,590	16,01	15,28	n.a.
3	Demethyl-Olea	10,787	0,179	1,222	3,79	7,21	n.a.
4	Olea	12,823	1,032	6,585	21,84	38,86	9,9239
5	Oleacein	13,860	0,034	0,296	0,73	1,74	n.a.
n.a.	Agiicone Olea	n.a.	n.a.	n.a.	n.a.	n.a.	n.a.
**Total:**			**4,723**	**16,946**	**100,00**	**100,00**	

NADES-3

Dried Leaves

Chromatogram and Results

Injection Details

Injection Name:	ChCL+Ac. Lattico (F.S.)_in 50 uL_03-12-18	*Run Time (min):*	35,00
Vial Number:	27	*Injection Volume:*	20,00
Injection Type:	Unknown	*Channel:*	UV_VIS_2
Calibration Level:		*Wavelength:*	280
Instrument Method:	Oleaolna MIX OK	*Bandwidth:*	2
Processing Method:	Prova	*Dilution Factor:*	1,0000
Injection Date/Time:	03/dic/18 14:27	*Sample Weight:*	1,0000

Chromatogram

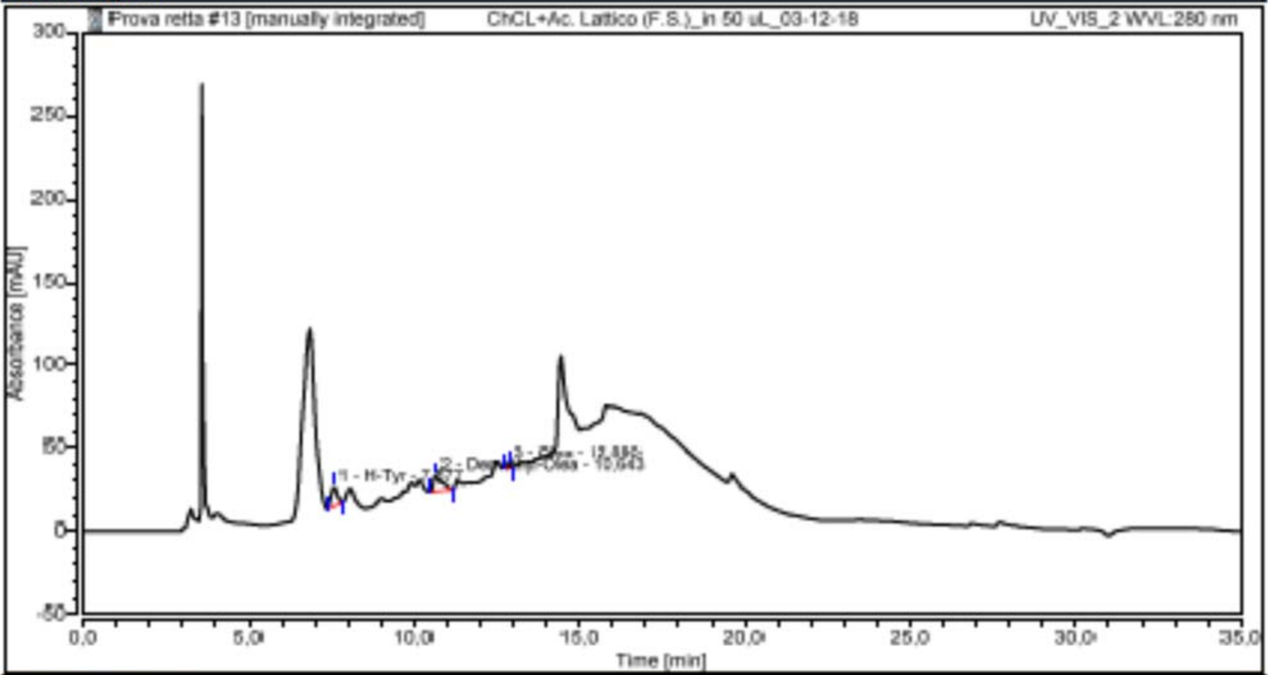

Integration Results

No.	Peak Name	Retention Time min	Area mAU*min	Height mAU	Relative Area %	Relative Height %	Amount
1	H-Tyr	7,577	2,487	11,455	45,87	51,11	n.a.
n.a.	Tyrosol	n.a.	n.a.	n.a.	n.a.	n.a.	n.a.
2	Demethyl-Olea	10,643	2,758	9,660	50,87	43,10	n.a.
3	Olea	12,890	0,177	1,299	3,26	5,80	2,5293
n.a.	Oleacein	n.a.	n.a.	n.a.	n.a.	n.a.	n.a.
n.a.	Aglicone Olea	n.a.	n.a.	n.a.	n.a.	n.a.	n.a.
**Total:**			**5,422**	**22,416**	**100,00**	**100,00**	

NADES-3

Fresh Leaves

Chromatogram and Results

Injection Details

Injection Name:	**ChCL+Ac. Lattico (F.V.)_in 50 uL_03-12-18**	*Run Time (min):*	**35,00**
Vial Number:	**29**	*Injection Volume:*	**20,00**
Injection Type:	**Unknown**	*Channel:*	**UV_VIS_2**
Calibration Level:		*Wavelength:*	**280**
Instrument Method:	**Oleaolna MIX OK**	*Bandwidth:*	**2**
Processing Method:	**Prova**	*Dilution Factor:*	**1,0000**
Injection Date/Time:	**03/dic/18 16:37**	*Sample Weight:*	**1,0000**

Chromatogram

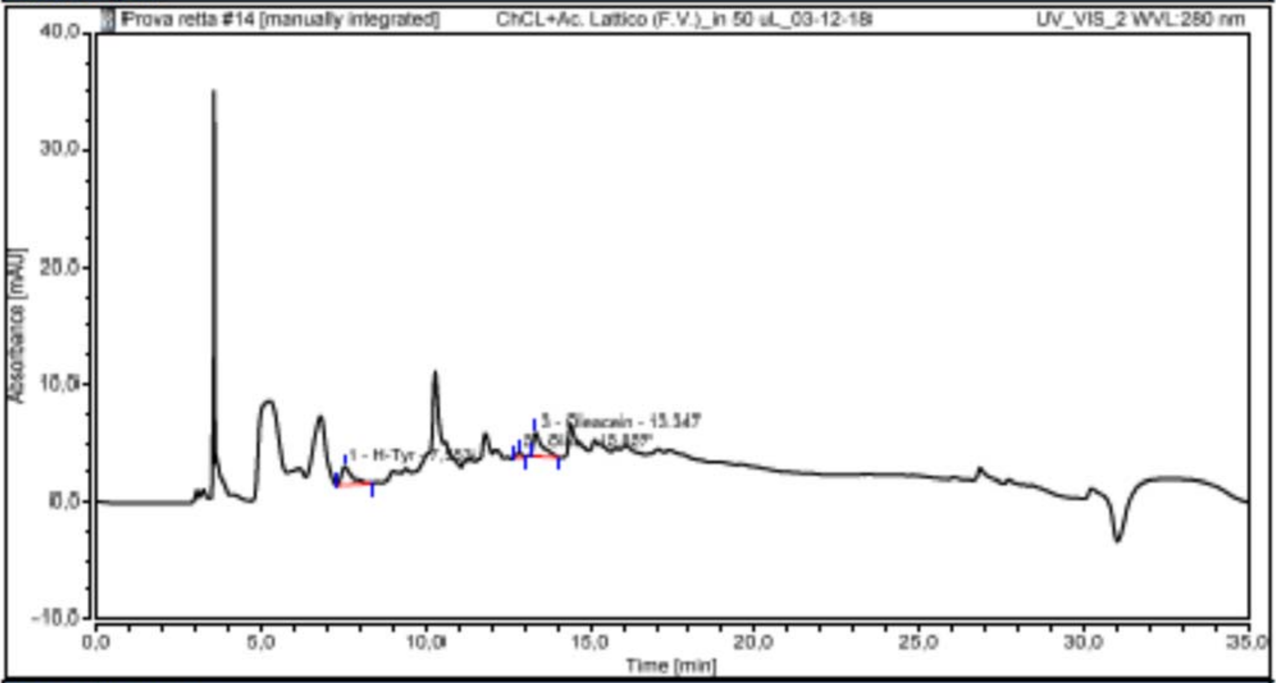

Integration Results

No.	Peak Name	Retention Time min	Area mAU*min	Height mAU	Relative Area %	Relative Height %	Amount
1	H-Tyr	7,553	0,559	1,553	43,30	39,14	n.a.
n.a.	Tyrosol	n.a.	n.a.	n.a.	n.a.	n.a.	n.a.
n.a.	Demethyl-Olea	n.a.	n.a.	n.a.	n.a.	n.a.	n.a.
2	Olea	12,827	0,081	0,404	6,25	10,18	1,6971
3	Oleacein	13,347	0,652	2,012	50,45	50,68	n.a.
n.a.	Aglicone Olea	n.a.	n.a.	n.a.	n.a.	n.a.	n.a.
**Total:**			**1,292**	**3,969**	**100,00**	**100,00**	

NADES-4

Dried Leaves

Chromatogram and Results

Injection Details			
Injection Name:	ChCl+Glicole-Et (F.S.)_in 50 uL_29-11-18	*Run Time (min):*	35,00
Vial Number:	22	*Injection Volume:*	20,00
Injection Type:	Unknown	*Channel:*	UV_VIS_2
Calibration Level:		*Wavelength:*	280
Instrument Method:	Oleaolna MIX OK	*Bandwidth:*	2
Processing Method:	Prova	*Dilution Factor:*	1,0000
Injection Date/Time:	29/nov/18 12:05	*Sample Weight:*	1,0000

Chromatogram

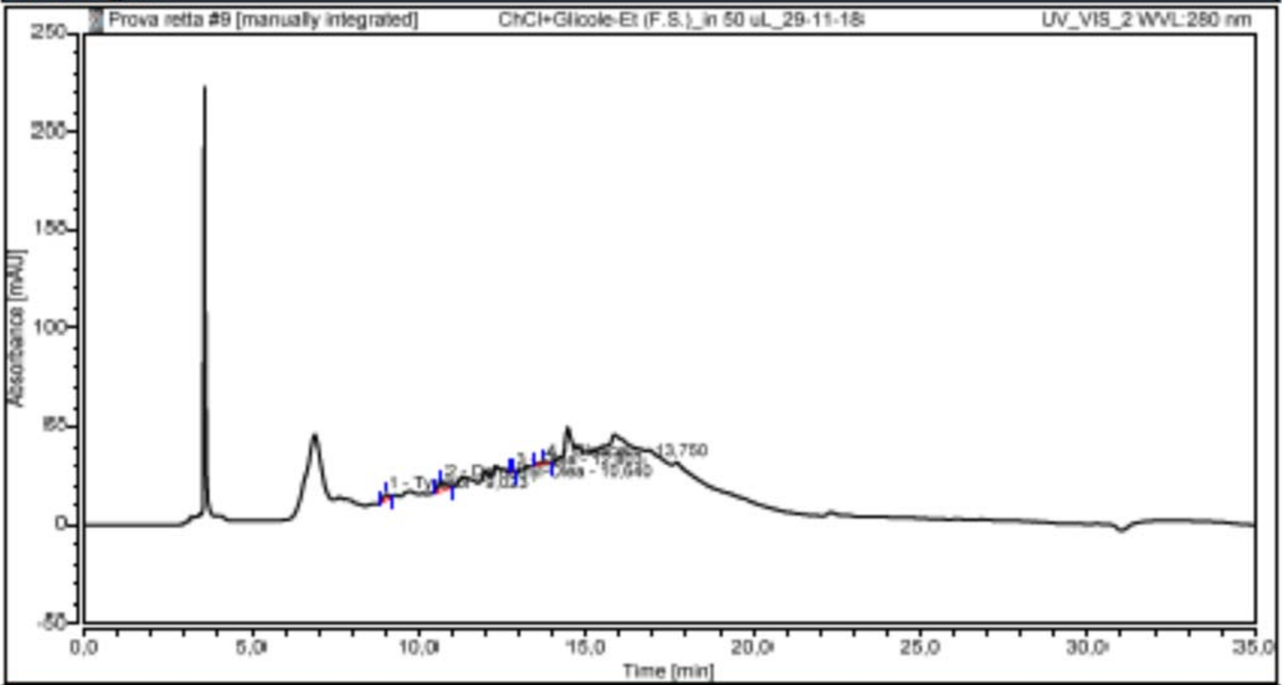

Integration Results

No.	Peak Name	Retention Time min	Area mAU*min	Height mAU	Relative Area %	Relative Height %	Amount
n.a.	H-Tyr	n.a.	n.a.	n.a.	n.a.	n.a.	n.a.
1	Tyrosol	9,023	0,542	3,158	24,07	32,44	n.a.
2	Demethyl-Olea	10,640	1,141	4,383	50,70	45,02	n.a.
3	Olea	12,803	0,082	0,721	3,66	7,40	1,7104
4	Oleacein	13,750	0,486	1,474	21,58	15,14	n.a.
n.a.	Aglicone Olea	n.a.	n.a.	n.a.	n.a.	n.a.	n.a.
**Total:**			2,251	9,736	100,00	100,00	

NADES-4

Fresh Leaves

Chromatogram and Results

Injection Details

Injection Name:	**ChCl+Glicole-Et (F.V.)_in 50uL_29-11-18**	*Run Time (min):*	**35,00**
Vial Number:	**22**	*Injection Volume:*	**20,00**
Injection Type:	**Unknown**	*Channel:*	**UV_VIS_2**
Calibration Level:		*Wavelength:*	**280**
Instrument Method:	**Oleaolna MIX OK**	*Bandwidth:*	**2**
Processing Method:	**Prova**	*Dilution Factor:*	**1,0000**
Injection Date/Time:	**29/nov/18 13:17**	*Sample Weight:*	**1,0000**

Chromatogram

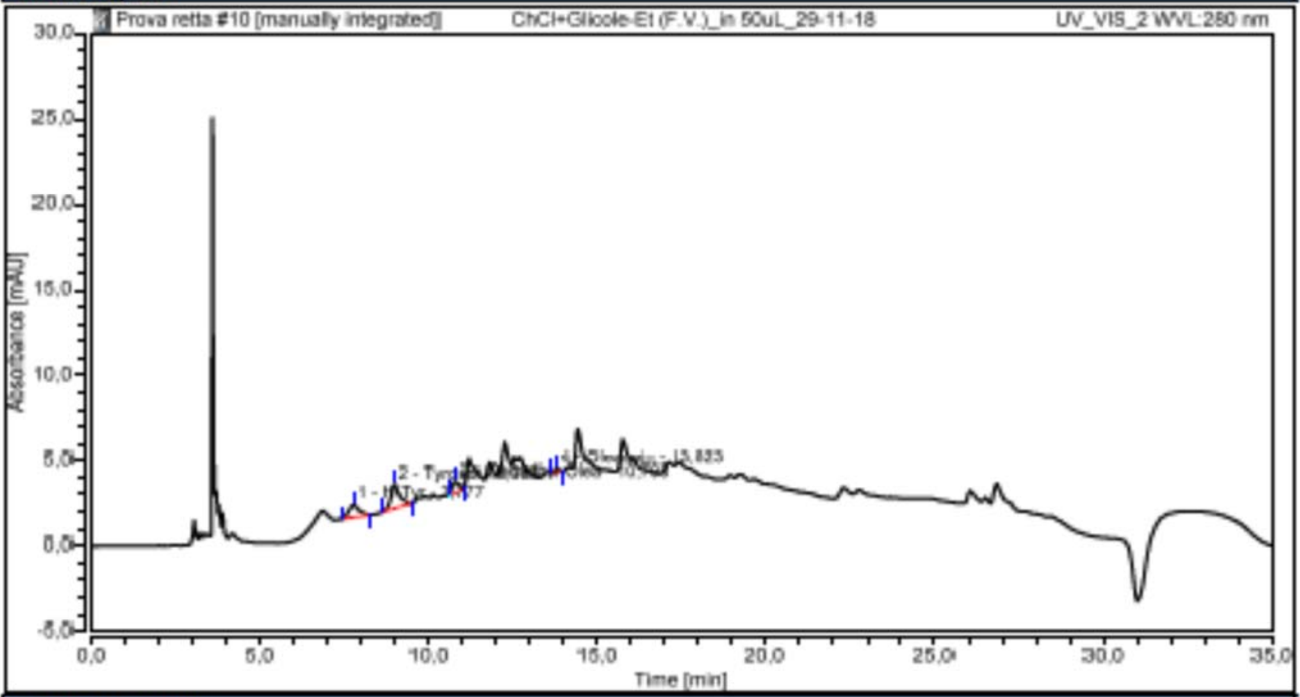

Integration Results

No.	Peak Name	Retention Time min	Area mAU*min	Height mAU	Relative Area %	Relative Height %	Amount
1	H-Tyr	7,777	0,259	0,762	29,51	24,60	n.a.
2	Tyrosol	8,983	0,421	1,441	48,06	46,54	n.a.
3	Demethyl-Olea	10,793	0,168	0,672	19,17	21,70	n.a.
n.a.	Olea	n.a.	n.a.	n.a.	n.a.	n.a.	n.a.
4	Oleacein	13,823	0,029	0,222	3,26	7,15	n.a.
n.a.	Aglicone Olea	n.a.	n.a.	n.a.	n.a.	n.a.	n.a.
**Total:**			**0,877**	**3,097**	**100,00**	**100,00**	

NADES-5

Dried Leaves

Chromatogram and Results

Injection Details

Injection Name:	**ChCl+Ac. Citrico (F.S.)_in 50 uL_29-11-18**	*Run Time (min):*	**35,00**
Vial Number:	**22**	*Injection Volume:*	**20,00**
Injection Type:	**Unknown**	*Channel:*	**UV_VIS_2**
Calibration Level:		*Wavelength:*	**280**
Instrument Method:	**Oleaolna MIX OK**	*Bandwidth:*	**2**
Processing Method:	**Prova**	*Dilution Factor:*	**1,0000**
Injection Date/Time:	**29/nov/18 14:43**	*Sample Weight:*	**1,0000**

Chromatogram

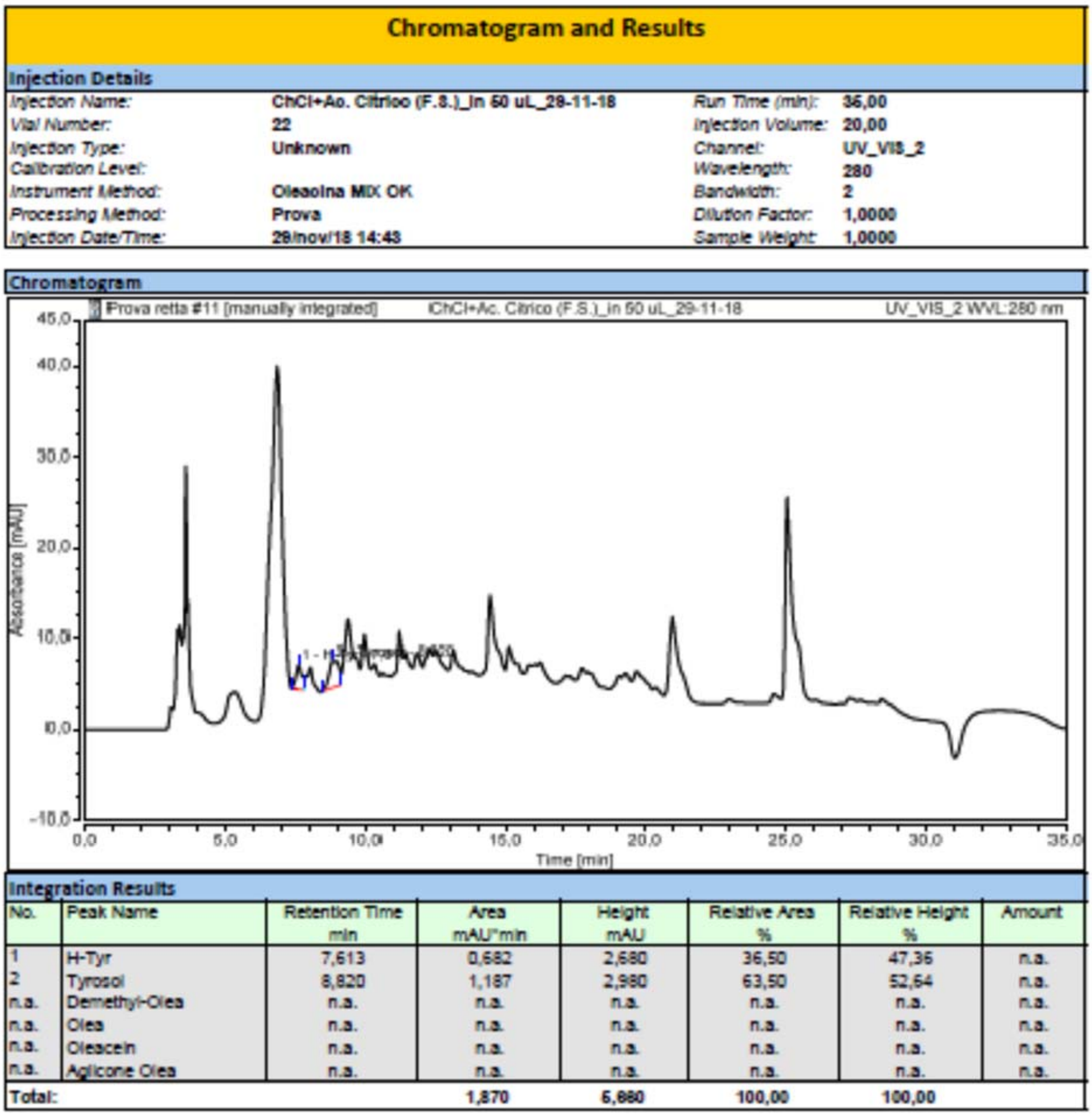

Integration Results

No.	Peak Name	Retention Time min	Area mAU*min	Height mAU	Relative Area %	Relative Height %	Amount
1	H-Tyr	7,613	0,682	2,680	36,50	47,36	n.a.
2	Tyrosol	8,820	1,187	2,980	63,50	52,64	n.a.
n.a.	Demethyl-Olea	n.a.	n.a.	n.a.	n.a.	n.a.	n.a.
n.a.	Olea	n.a.	n.a.	n.a.	n.a.	n.a.	n.a.
n.a.	Oleacein	n.a.	n.a.	n.a.	n.a.	n.a.	n.a.
n.a.	Aglicone Olea	n.a.	n.a.	n.a.	n.a.	n.a.	n.a.
**Total:**			**1,870**	**5,660**	**100,00**	**100,00**	

NADES-5

Fresh Leaves

Chromatogram and Results

Injection Details

Injection Name:	ChCl+Ac. Citrico (F.V.)_in 50uL_29-11-18	*Run Time (min):*	35,00
Vial Number:	22	*Injection Volume:*	20,00
Injection Type:	Unknown	*Channel:*	UV_VIS_2
Calibration Level:		*Wavelength:*	280
Instrument Method:	Oleaoina MIX OK	*Bandwidth:*	2
Processing Method:	Prova	*Dilution Factor:*	1,0000
Injection Date/Time:	29/nov/18 16:04	*Sample Weight:*	1,0000

Chromatogram

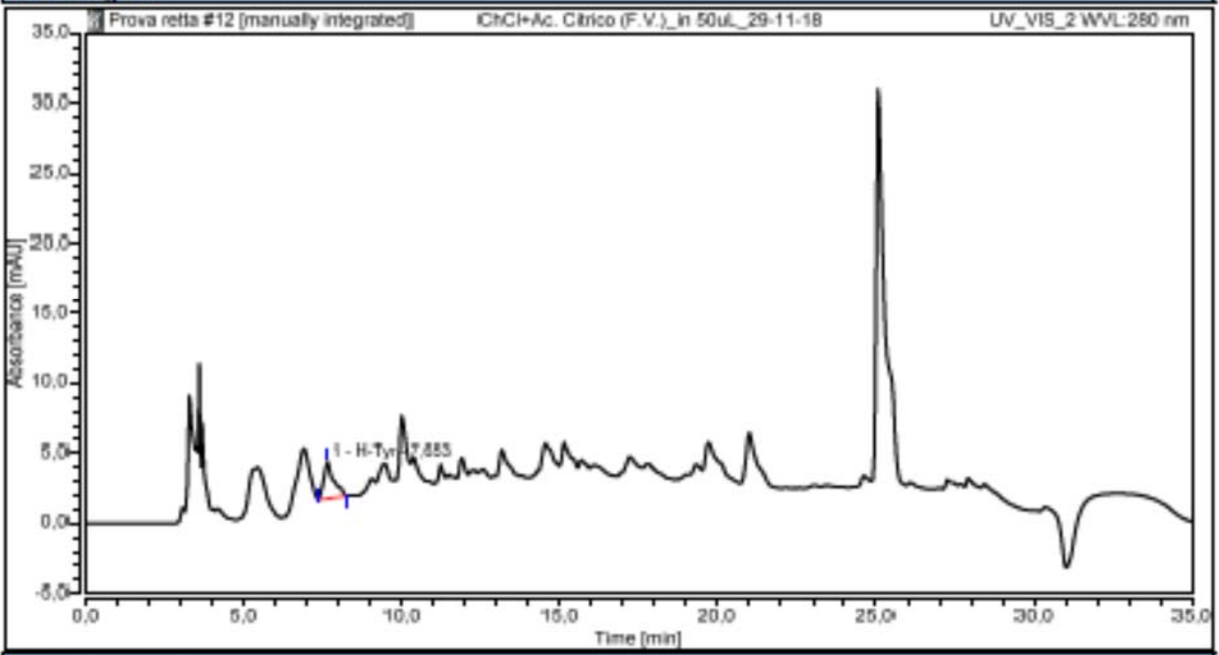

Integration Results

No.	Peak Name	Retention Time min	Area mAU*min	Height mAU	Relative Area %	Relative Height %	Amount
1	H-Tyr	7,653	0,951	2,640	100,00	100,00	n.a.
n.a.	Tyrosol	n.a.	n.a.	n.a.	n.a.	n.a.	n.a.
n.a.	Demethyl-Olea	n.a.	n.a.	n.a.	n.a.	n.a.	n.a.
n.a.	Olea	n.a.	n.a.	n.a.	n.a.	n.a.	n.a.
n.a.	Oleacein	n.a.	n.a.	n.a.	n.a.	n.a.	n.a.
n.a.	Aglicone Olea	n.a.	n.a.	n.a.	n.a.	n.a.	n.a.
**Total:**			0,951	2,640	100,00	100,00	

Water

Dried Leaves

Chromatogram and Results

Injection Details

Injection Name:	**H2O (F.S.)_in 50 uL_04-12-18**	*Run Time (min):*	**35,00**
Vial Number:	**31**	*Injection Volume:*	**20,00**
Injection Type:	**Unknown**	*Channel:*	**UV_VIS_2**
Calibration Level:		*Wavelength:*	**280**
Instrument Method:	**Oleaolna MIX OK**	*Bandwidth:*	**2**
Processing Method:	**Prova**	*Dilution Factor:*	**1,0000**
Injection Date/Time:	**04/dic/18 18:24**	*Sample Weight:*	**1,0000**

Chromatogram

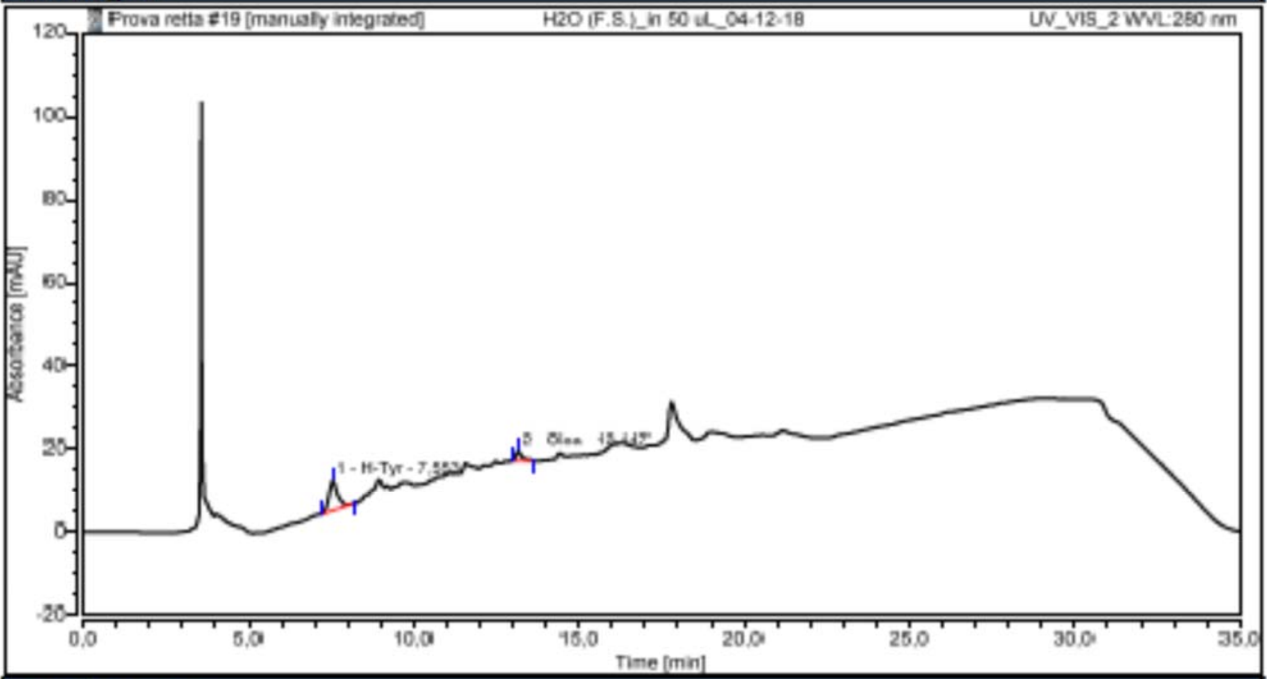

Integration Results

No.	Peak Name	Retention Time<br>min	Area<br>mAU*min	Height<br>mAU	Relative Area<br>%	Relative Height<br>%	Amount
1	H-Tyr	7,553	2,170	7,302	81,69	76,06	n.a.
n.a.	Tyrosol	n.a.	n.a.	n.a.	n.a.	n.a.	n.a.
n.a.	Demethyl-Olea	n.a.	n.a.	n.a.	n.a.	n.a.	n.a.
2	Olea	13,147	0,486	2,298	18,31	23,94	5,2061
n.a.	Oleacein	n.a.	n.a.	n.a.	n.a.	n.a.	n.a.
n.a.	Aglicone Olea	n.a.	n.a.	n.a.	n.a.	n.a.	n.a.
**Total:**			**2,656**	**9,600**	**100,00**	**100,00**	

Water

Fresh Leaves

Chromatogram and Results

Injection Details

Injection Name:	**H2O (F.V.)_In 50 uL_04-12-18**	*Run Time (min):*	**35,00**
Vial Number:	**31**	*Injection Volume:*	**20,00**
Injection Type:	**Unknown**	*Channel:*	**UV_VIS_2**
Calibration Level:		*Wavelength:*	**280**
Instrument Method:	**Oleaoina MIX OK**	*Bandwidth:*	**2**
Processing Method:	**Prova**	*Dilution Factor:*	**1,0000**
Injection Date/Time:	**04/dic/18 17:35**	*Sample Weight:*	**1,0000**

Chromatogram

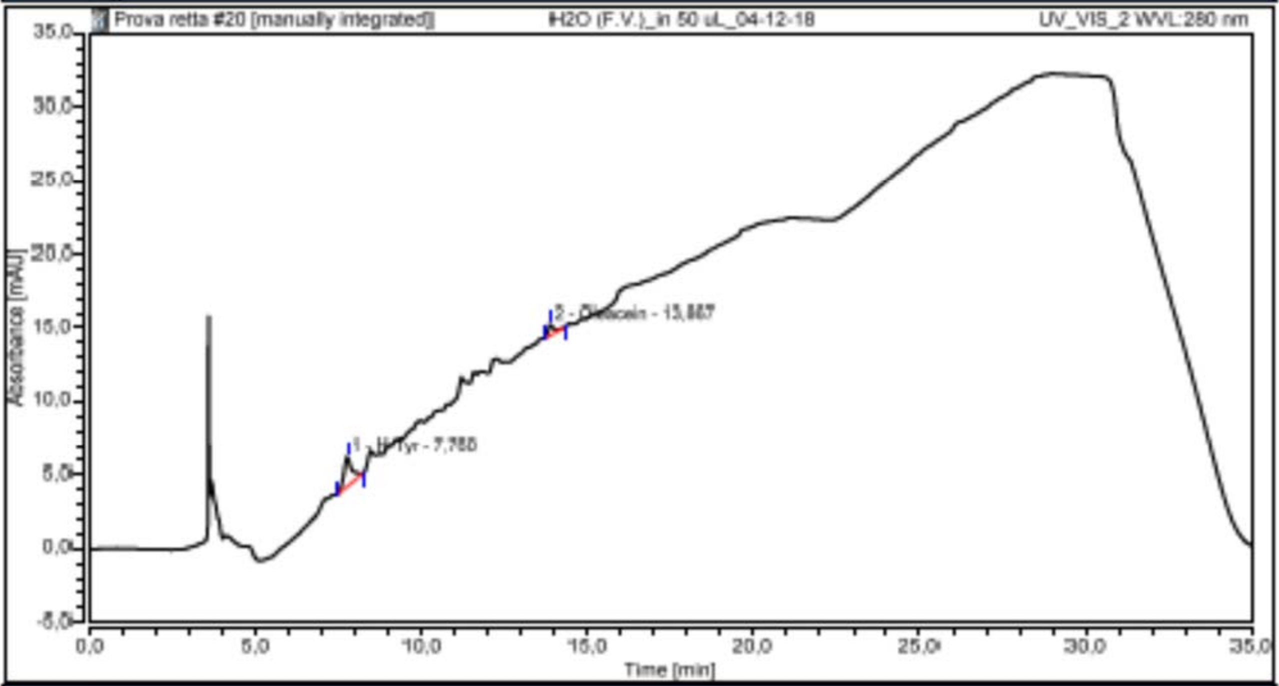

Integration Results

No.	Peak Name	Retention Time min	Area mAU*min	Height mAU	Relative Area %	Relative Height %	Amount
1	H-Tyr	7,760	0,563	2,043	79,36	72,95	n.a.
n.a.	Tyrosol	n.a.	n.a.	n.a.	n.a.	n.a.	n.a.
n.a.	Demethyl-Olea	n.a.	n.a.	n.a.	n.a.	n.a.	n.a.
n.a.	Olea	n.a.	n.a.	n.a.	n.a.	n.a.	n.a.
2	Oleacein	13,867	0,146	0,758	20,64	27,05	n.a.
n.a.	Aglicone Olea	n.a.	n.a.	n.a.	n.a.	n.a.	n.a.
**Total:**			**0,710**	**2,801**	**100,00**	**100,00**	

NADES-1

Ripe Olives (10 min.)

Chromatogram and Results

Injection Details

Injection Name:	Grezzo DES 1_10 min_22-06-18	*Run Time (min):*	35,00
Vial Number:	12	*Injection Volume:*	20,00
Injection Type:	Unknown	*Channel:*	UV_VIS_2
Calibration Level:		*Wavelength:*	280
Instrument Method:	Oleacina MIX OK	*Bandwidth:*	2
Processing Method:	Quantificazione Oleacina Mix	*Dilution Factor:*	1,0000
Injection Date/Time:	22/giu/18 11:34	*Sample Weight:*	1,0000

Chromatogram

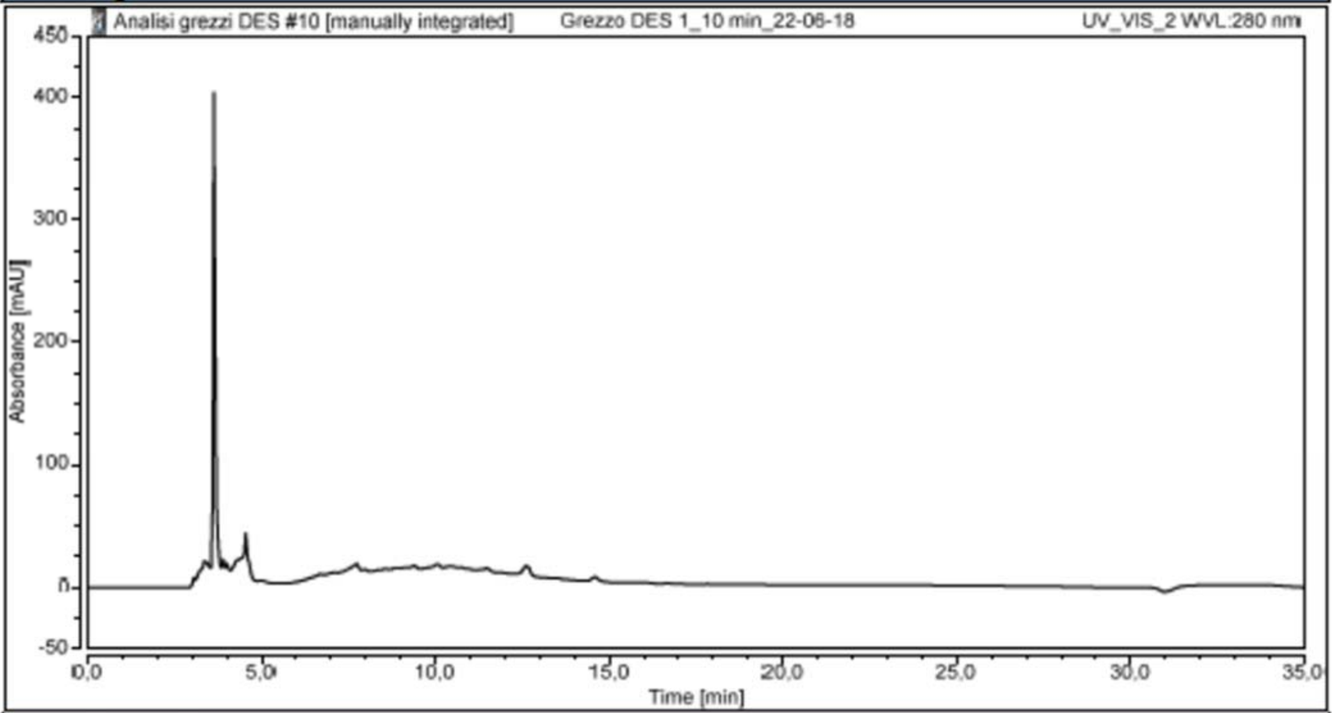

Integration Results

No.	Peak Name	Retention Time min	Area mAU*min	Height mAU	Relative Area %	Relative Height %	Amount ppm
n.a.	Htyr	n.a.	n.a.	n.a.	n.a.	n.a.	n.a.
n.a.	Demethylolea	n.a.	n.a.	n.a.	n.a.	n.a.	n.a.
n.a.	Olea	n.a.	n.a.	n.a.	n.a.	n.a.	n.a.
n.a.	Oleacina	n.a.	n.a.	n.a.	n.a.	n.a.	n.a.
n.a.	Aglicone Olea	n.a.	n.a.	n.a.	n.a.	n.a.	n.a.
Total:			0,000	0,000	0,00	0,00	

NADES-1

Ripe Olives (30 min.)

Chromatogram and Results

Injection Details

Injection Name:	Grezzo DES 1_30 min_26-06-18	*Run Time (min):*	35,00
Vial Number:	11	*Injection Volume:*	20,00
Injection Type:	Unknown	*Channel:*	UV_VIS_2
Calibration Level:		*Wavelength:*	280
Instrument Method:	Oleacina MIX OK	*Bandwidth:*	2
Processing Method:	Quantificazione Oleacina Mix	*Dilution Factor:*	1,0000
Injection Date/Time:	26/giu/18 11:41	*Sample Weight:*	1,0000

Chromatogram

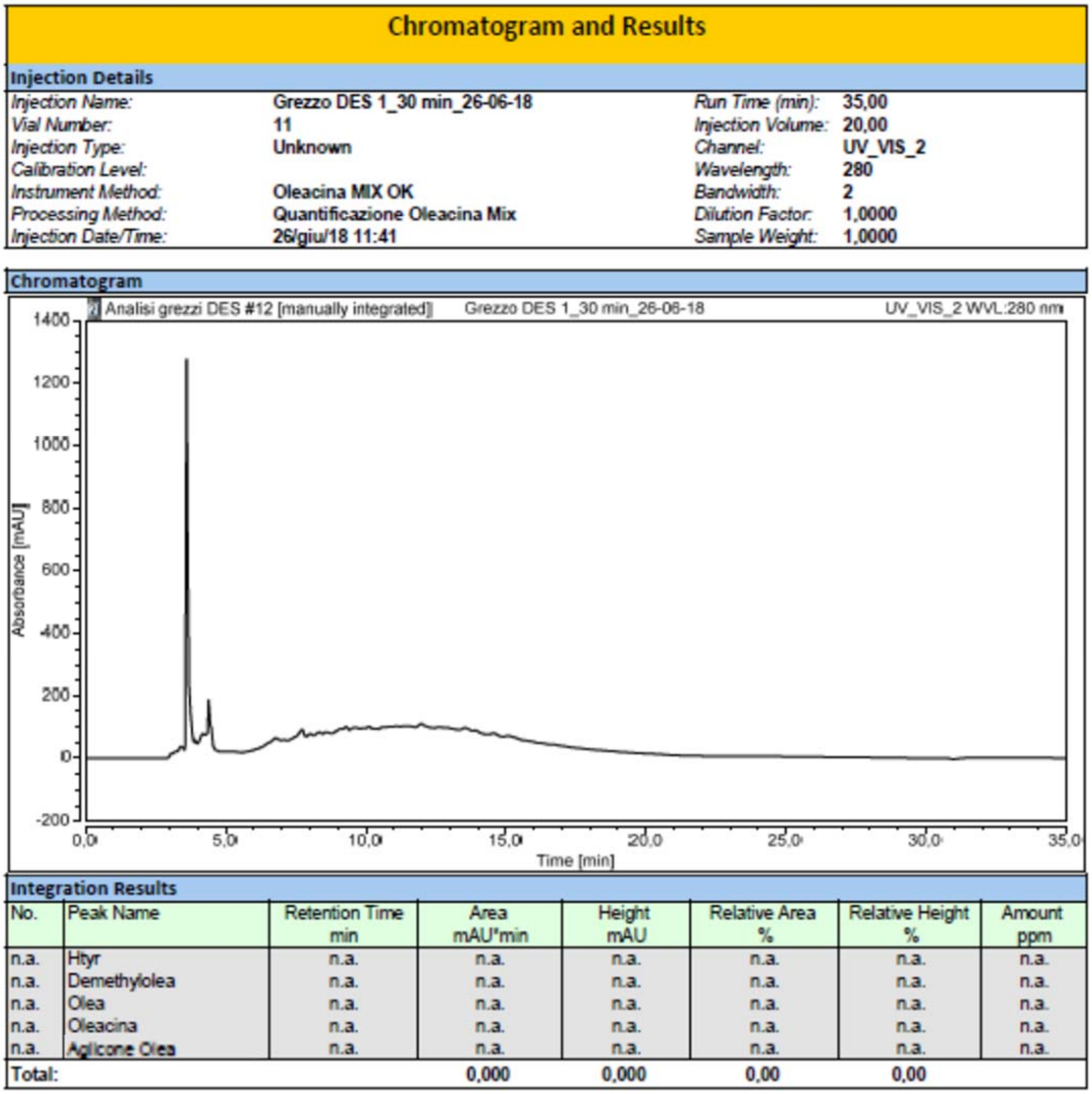

Integration Results

No.	Peak Name	Retention Time min	Area mAU*min	Height mAU	Relative Area %	Relative Height %	Amount ppm
n.a.	Htyr	n.a.	n.a.	n.a.	n.a.	n.a.	n.a.
n.a.	Demethylolea	n.a.	n.a.	n.a.	n.a.	n.a.	n.a.
n.a.	Olea	n.a.	n.a.	n.a.	n.a.	n.a.	n.a.
n.a.	Oleacina	n.a.	n.a.	n.a.	n.a.	n.a.	n.a.
n.a.	Aglicone Olea	n.a.	n.a.	n.a.	n.a.	n.a.	n.a.
Total:			0,000	0,000	0,00	0,00	

NADES-2

Ripe Olives (10 min.)

Chromatogram and Results

Injection Details

Injection Name:	**Grezzo DES 2_10 min_27-06-18**	*Run Time (min):*	**36,00**
Vial Number:	**17**	*Injection Volume:*	**20,00**
Injection Type:	**Unknown**	*Channel:*	**UV_VIS_2**
Calibration Level:		*Wavelength:*	**280**
Instrument Method:	**Oleaolna MIX OK**	*Bandwidth:*	**2**
Processing Method:	**Quantificazione Oleaolna Mix**	*Dilution Factor:*	**1,0000**
Injection Date/Time:	**27/giu/18 11:29**	*Sample Weight:*	**1,0000**

Chromatogram

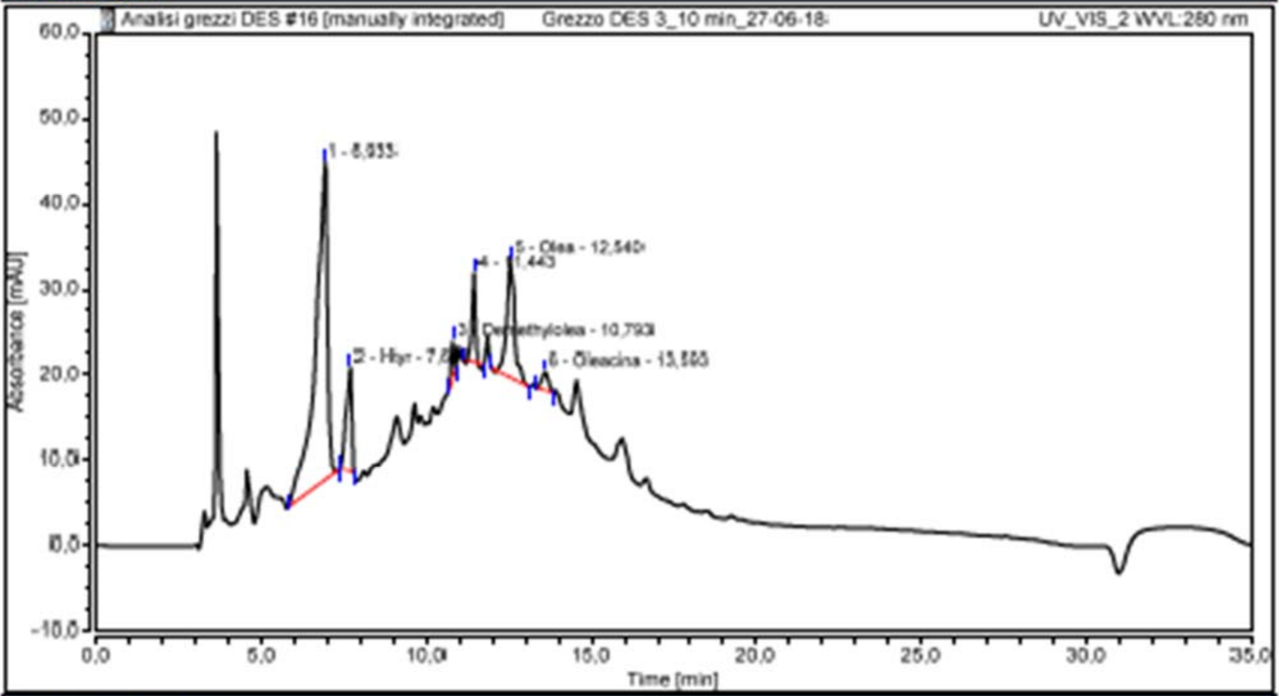

Integration Results

No.	Peak Name	Retention Time min	Area mAU*min	Height mAU	Relative Area %	Relative Height %	Amount ppm
1		6,933	15,330	37,186	65,25	46,54	n.a.
2	Htyr	7,693	2,350	12,041	10,00	15,07	n.a.
3	Demethyloleа	10,793	0,464	4,395	1,98	5,50	7,3206
4		11,443	1,144	10,402	4,87	13,02	n.a.
5	Olea	12,540	3,722	13,720	15,84	17,17	2270,2518
6	Oleacina	13,593	0,484	2,156	2,06	2,70	4,0279
n.a.	Aglicone Olea	n.a.	n.a.	n.a.	n.a.	n.a.	n.a.
Total:			0,000	0,000	0,00	0,00	

NADES-2

Ripe Olives (30 min.)

Chromatogram and Results

Injection Details

Injection Name:	**Grezzo DES 2_30 min_27-06-18**	*Run Time (min):*	**36,00**
Vial Number:	**17**	*Injection Volume:*	**20,00**
Injection Type:	**Unknown**	*Channel:*	**UV_VIS_2**
Calibration Level:		*Wavelength:*	**280**
Instrument Method:	**Oleaolna MIX OK**	*Bandwidth:*	**2**
Processing Method:	**Quantificazione Oleaolna Mix**	*Dilution Factor:*	**1,0000**
Injection Date/Time:	**27/giu/18 12:38**	*Sample Weight:*	**1,0000**

Chromatogram

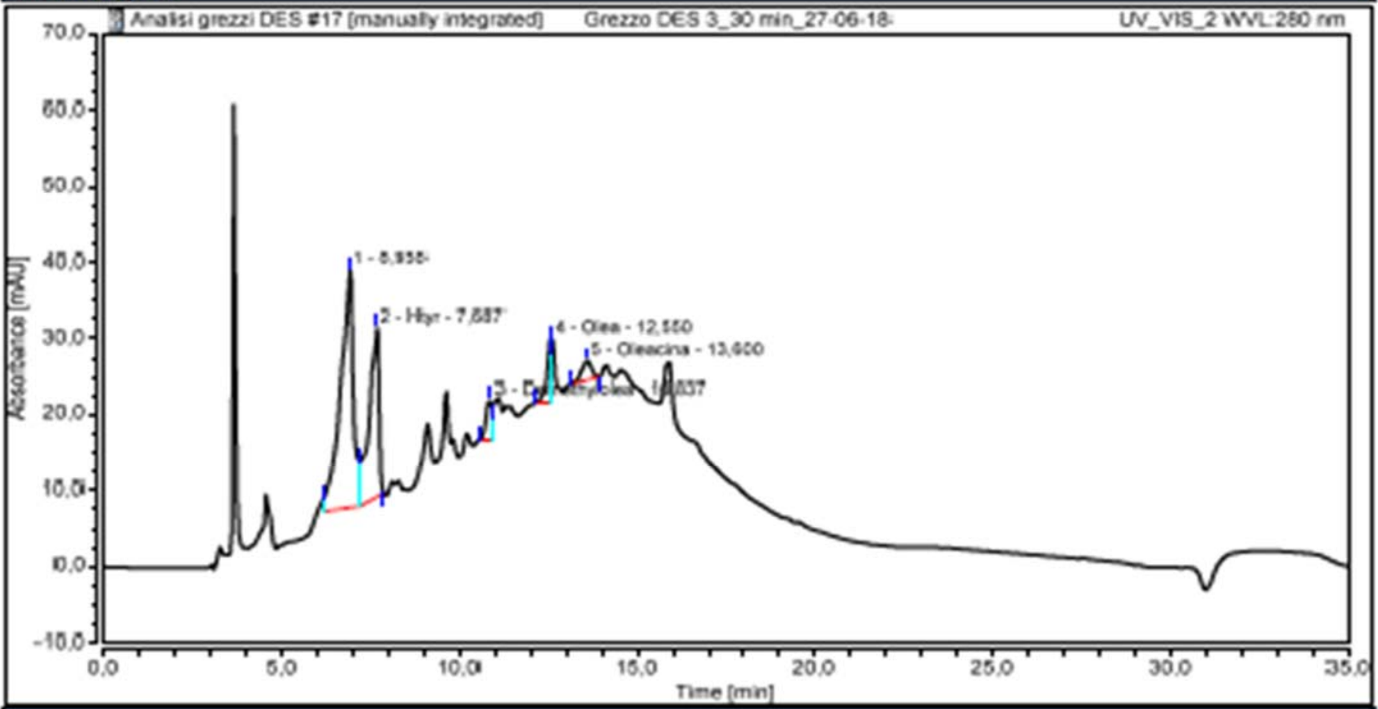

Integration Results

No.	Peak Name	Retention Time<br>min	Area<br>mAU*min	Height<br>mAU	Relative Area<br>%	Relative Height<br>%	Amount<br>ppm
1		6,930	13,059	31,155	56,36	44,82	n.a.
2	Htyr	7,687	7,128	22,223	30,77	31,97	n.a.
3	Demethyloiea	10,837	0,924	5,096	3,99	7,33	14,5692
4	Olea	12,550	1,233	8,485	5,32	12,21	752,0484
5	Oleacina	13,600	0,825	2,552	3,56	3,67	6,8686
n.a.	Aglicone Olea	n.a.	n.a.	n.a.	n.a.	n.a.	n.a.
Total:			**23,168**	**69,511**	**100,00**	**100,00**	

NADES-3

Ripe Olives (10 min.)

Chromatogram and Results

Injection Details

Injection Name:	**Grezzo DES 3_10 min_27-06-18**	*Run Time (min):*	**35,00**
Vial Number:	**17**	*Injection Volume:*	**20,00**
Injection Type:	**Unknown**	*Channel:*	**UV_VIS_2**
Calibration Level:		*Wavelength:*	**280**
Instrument Method:	**Oleaolna MIX OK**	*Bandwidth:*	**2**
Processing Method:	**Quantificazione Oleacina Mix**	*Dilution Factor:*	**1,0000**
Injection Date/Time:	**27/giu/18 16:17**	*Sample Weight:*	**1,0000**

Chromatogram

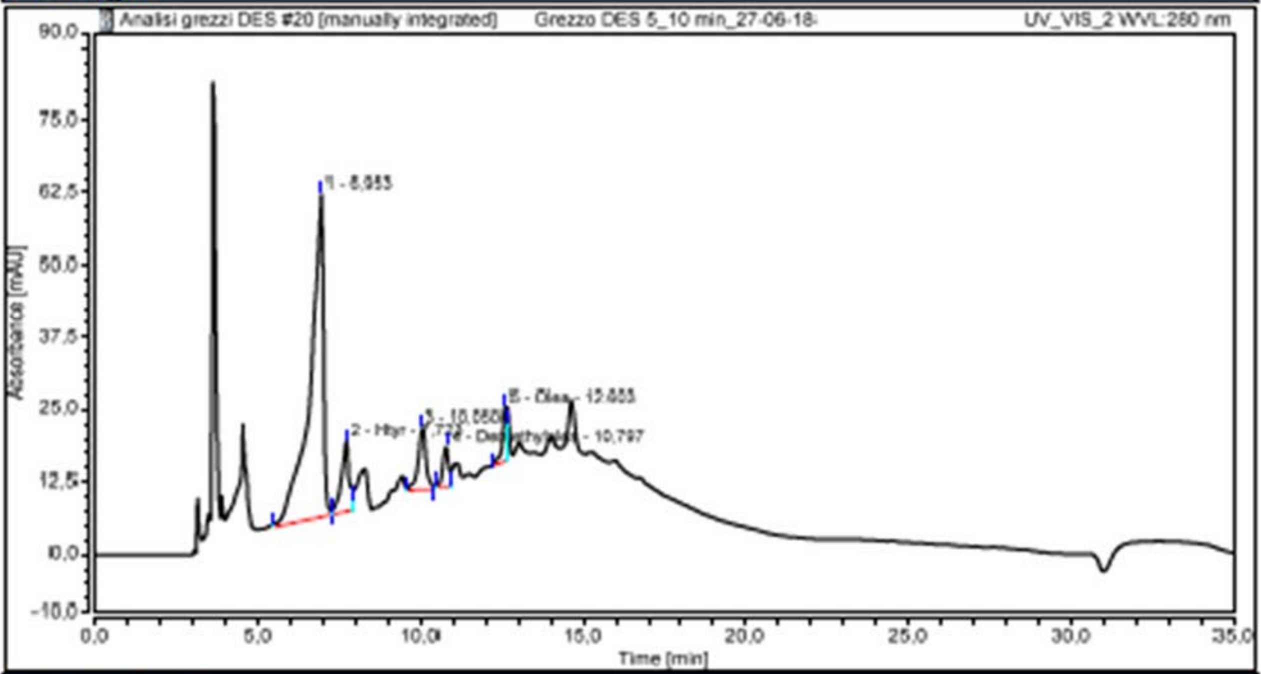

Integration Results

No.	Peak Name	Retention Time min	Area mAU*min	Height mAU	Relative Area %	Relative Height %	Amount ppm
1		6,953	25,926	55,721	74,48	59,00	n.a.
2	Htyr	7,723	3,230	12,080	9,28	12,79	n.a.
3		10,050	3,023	10,574	8,69	11,20	n.a.
4	Demethyloleа	10,797	1,391	6,833	4,00	7,23	21,9287
5	Olea	12,603	1,237	9,238	3,55	9,78	754,6103
n.a.	Oleacina	n.a.	n.a.	n.a.	n.a.	n.a.	n.a.
n.a.	Aglicone Olea	n.a.	n.a.	n.a.	n.a.	n.a.	n.a.
Total:			34,807	94,446	100,00	100,00	

NADES-3

Ripe Olives (30 min.)

Chromatogram and Results

Injection Details

Injection Name:	Grezzo DES 3_30 min_28-06-18	*Run Time (min):*	36,00
Vial Number:	17	*Injection Volume:*	20,00
Injection Type:	Unknown	*Channel:*	UV_VIS_2
Calibration Level:		*Wavelength:*	280
Instrument Method:	Oleaoina MIX OK	*Bandwidth:*	2
Processing Method:	Quantificazione Oleaoina Mix	*Dilution Factor:*	1,0000
Injection Date/Time:	28/giu/18 11:32	*Sample Weight:*	1,0000

Chromatogram

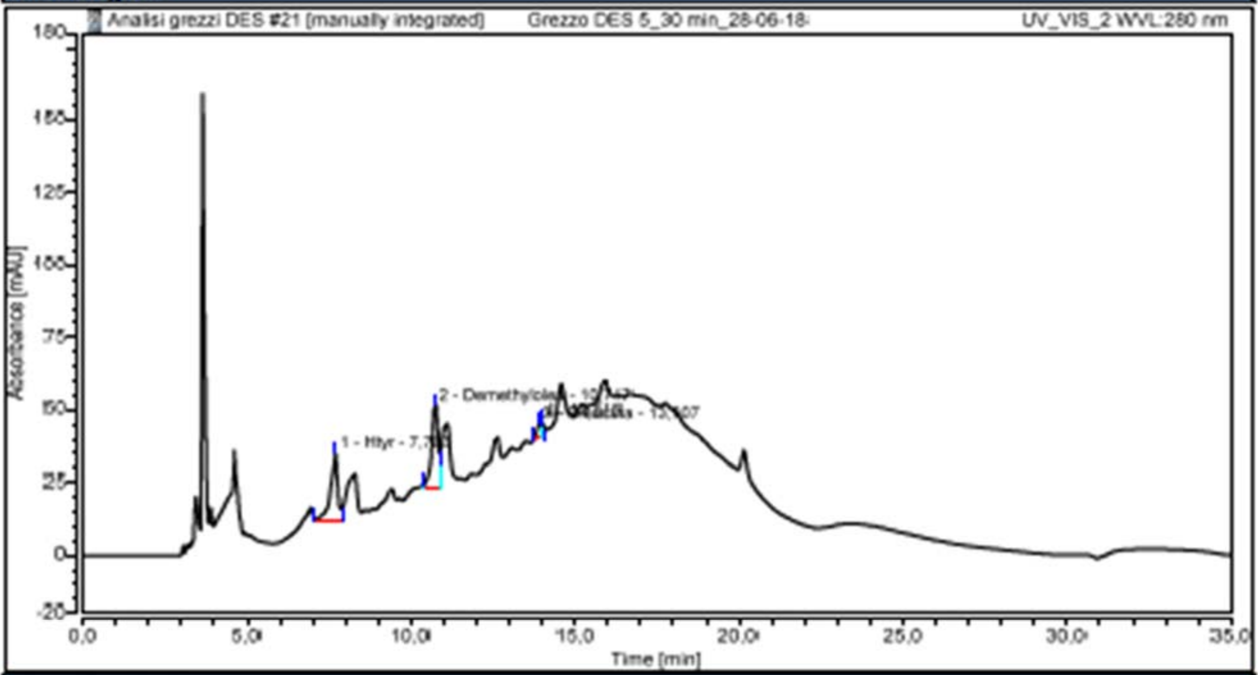

Integration Results

No.	Peak Name	Retention Time<br>min	Area<br>mAU*min	Height<br>mAU	Relative Area<br>%	Relative Height<br>%	Amount<br>ppm
1	Htyr	7,703	5,436	22,411	42,30	38,15	n.a.
2	Demethyloleuropein	10,717	6,398	27,822	49,79	47,36	100,8890
n.a.	Olea	n.a.	n.a.	n.a.	n.a.	n.a.	n.a.
3	Oleacina	13,907	0,516	4,137	4,02	7,04	4,3015
4	Aglicone Olea	14,010	0,499	4,371	3,89	7,44	n.a.
**Total:**			12,849	68,742	100,00	100,00	

NADES-4

Ripe Olives (10 min.)

Chromatogram and Results

Injection Details

Injection Name:	Grezzo DES 4_10 min_28-06-18	*Run Time (min):*	35,00
Vial Number:	17	*Injection Volume:*	20,00
Injection Type:	Unknown	*Channel:*	UV_VIS_2
Calibration Level:		*Wavelength:*	280
Instrument Method:	Oleacina MIX OK	*Bandwidth:*	2
Processing Method:	Quantificazione Oleacina Mix	*Dilution Factor:*	1,0000
Injection Date/Time:	28/giu/18 15:14	*Sample Weight:*	1,0000

Chromatogram

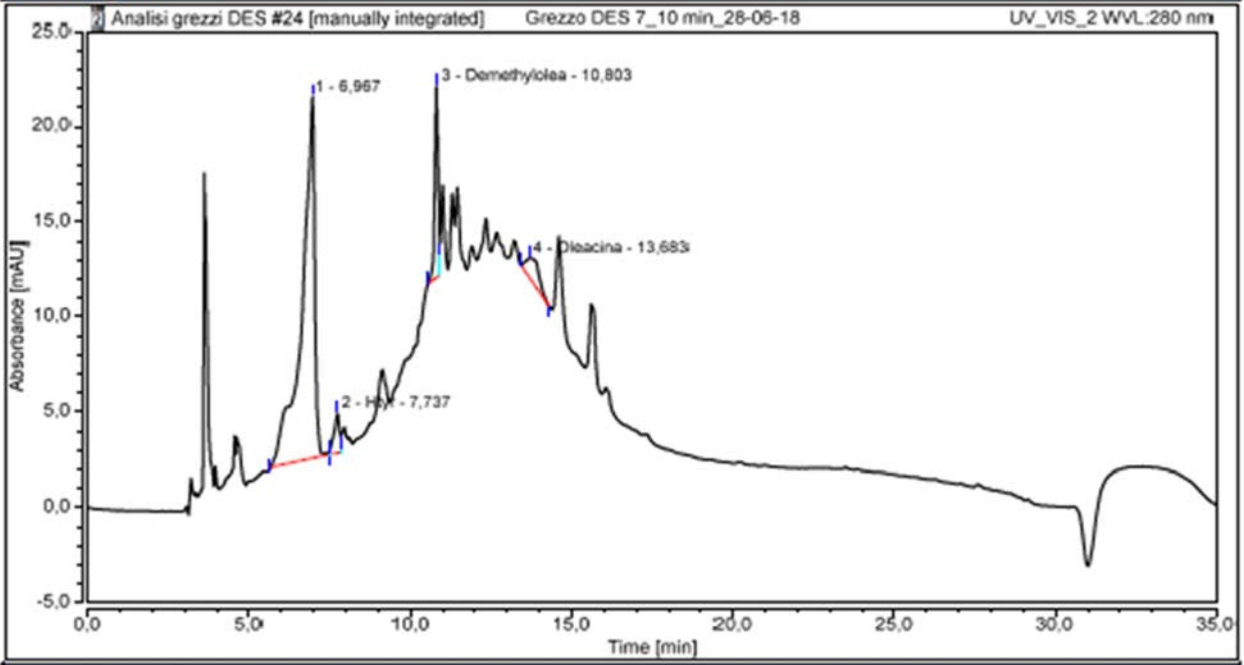

Integration Results

No.	Peak Name	Retention Time min	Area mAU*min	Height mAU	Relative Area %	Relative Height %	Amount ppm
1		6,967	8,609	19,003	79,75	59,16	n.a.
2	Htyr	7,737	0,408	2,062	3,78	6,42	n.a.
3	Demethyloleа	10,803	1,200	10,049	11,12	31,28	18,9254
n.a.	Olea	n.a.	n.a.	n.a.	n.a.	n.a.	n.a.
4	Oleacina	13,683	0,578	1,010	5,35	3,14	4,8107
n.a.	Aglicone Olea	n.a.	n.a.	n.a.	n.a.	n.a.	n.a.
Total:			10,795	32,124	100,00	100,00	

NADES-4

Ripe Olives (30 min.)

Chromatogram and Results

Injection Details

Injection Name:	Grezzo DES 4_30 min_28-06-18	*Run Time (min):*	35,00
Vial Number:	17	*Injection Volume:*	20,00
Injection Type:	Unknown	*Channel:*	UV_VIS_2
Calibration Level:		*Wavelength:*	280
Instrument Method:	Oleacina MIX OK	*Bandwidth:*	2
Processing Method:	Quantificazione Oleacina Mix	*Dilution Factor:*	1,0000
Injection Date/Time:	28/giu/18 16:27	*Sample Weight:*	1,0000

Chromatogram

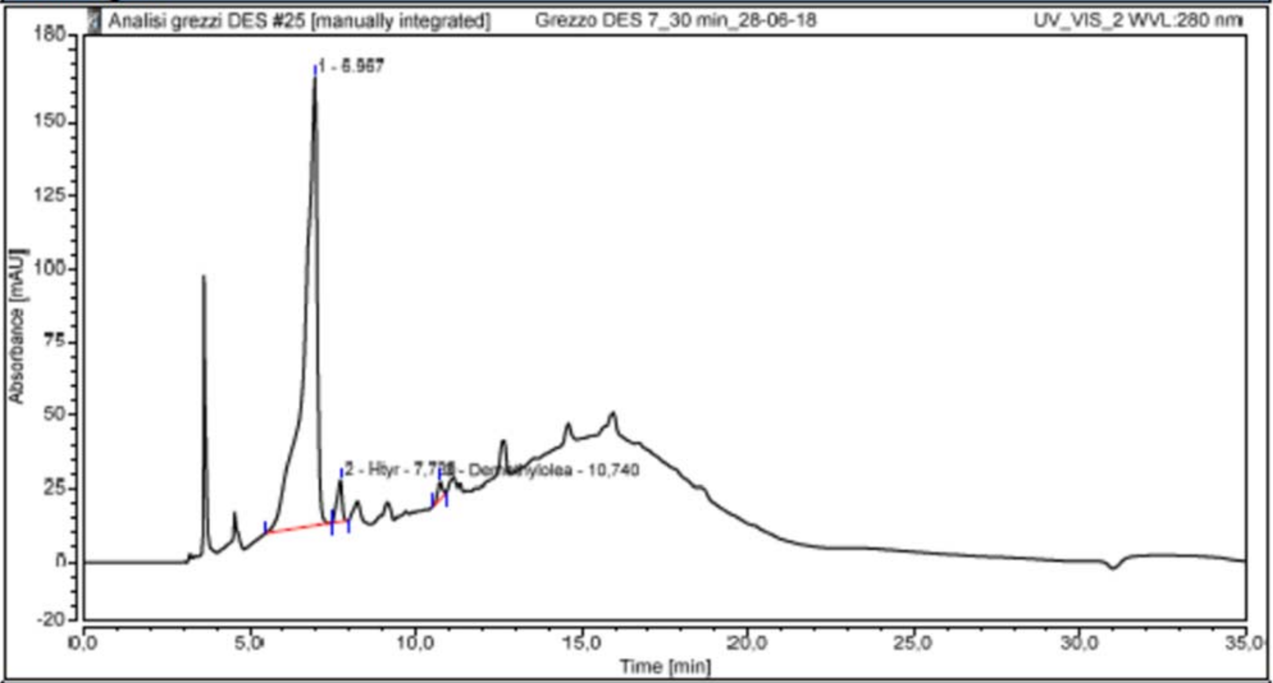

Integration Results

No.	Peak Name	Retention Time min	Area mAU*min	Height mAU	Relative Area %	Relative Height %	Amount ppm
1		6,967	68,641	153,044	95,18	88,55	n.a.
2	Htyr	7,730	2,527	14,004	3,50	8,10	n.a.
3	Demethylolea	10,740	0,950	5,788	1,32	3,35	14,9771
n.a.	Olea	n.a.	n.a.	n.a.	n.a.	n.a.	n.a.
n.a.	Oleacina	n.a.	n.a.	n.a.	n.a.	n.a.	n.a.
n.a.	Aglicone Olea	n.a.	n.a.	n.a.	n.a.	n.a.	n.a.
**Total:**			**72,118**	**172,835**	**100,00**	**100,00**	

NADES-5

Ripe Olives (10 min.)

Chromatogram and Results

Injection Details

Injection Name:	Grezzo DES 5_10 min_02-07-18	*Run Time (min):*	35,00
Vial Number:	11	*Injection Volume:*	20,00
Injection Type:	Unknown	*Channel:*	UV_VIS_2
Calibration Level:		*Wavelength:*	280
Instrument Method:	Oleacina MIX OK	*Bandwidth:*	2
Processing Method:	Quantificazione Oleacina Mix	*Dilution Factor:*	1,0000
Injection Date/Time:	02/lug/18 10:47	*Sample Weight:*	1,0000

Chromatogram

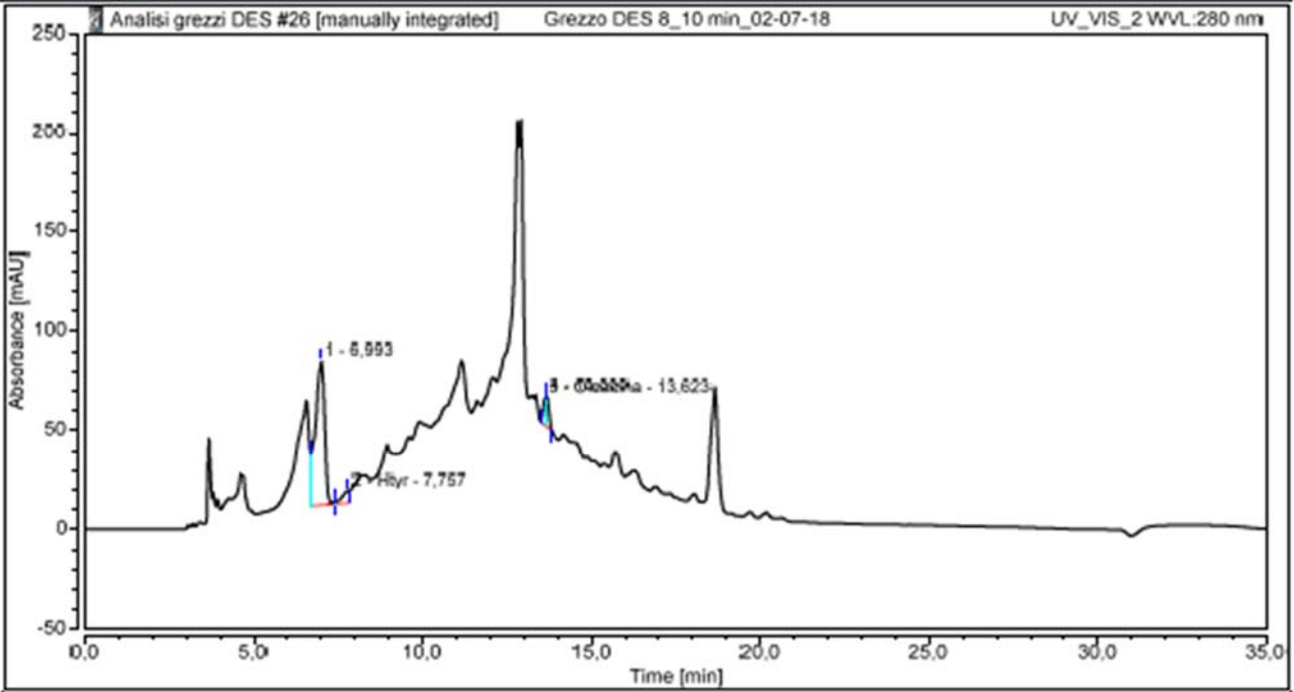

Integration Results

No.	Peak Name	Retention Time min	Area mAU*min	Height mAU	Relative Area %	Relative Height %	Amount ppm
1		6,993	23,750	72,915	84,92	67,40	n.a.
2	Htyr	7,757	1,457	6,107	5,21	5,65	n.a.
n.a.	Demethylolea	n.a.	n.a.	n.a.	n.a.	n.a.	n.a.
n.a.	Olea	n.a.	n.a.	n.a.	n.a.	n.a.	n.a.
3	Oleacina	13,623	1,267	13,840	4,53	12,79	10,5557
4	Aglicone Olea	13,690	1,492	15,314	5,34	14,16	n.a.
**Total:**			**27,966**	**108,176**	**100,00**	**100,00**	

NADES-5

Ripe Olives (30 min.)

Chromatogram and Results

Injection Details

Injection Name:	Grezzo DES 5_30 min_02-07-18	*Run Time (min):*	35,00
Vial Number:	13	*Injection Volume:*	20,00
Injection Type:	Unknown	*Channel:*	UV_VIS_2
Calibration Level:		*Wavelength:*	280
Instrument Method:	Oleacina MIX OK	*Bandwidth:*	2
Processing Method:	Quantificazione Oleacina Mix	*Dilution Factor:*	1,0000
Injection Date/Time:	02/lug/18 12:03	*Sample Weight:*	1,0000

Chromatogram

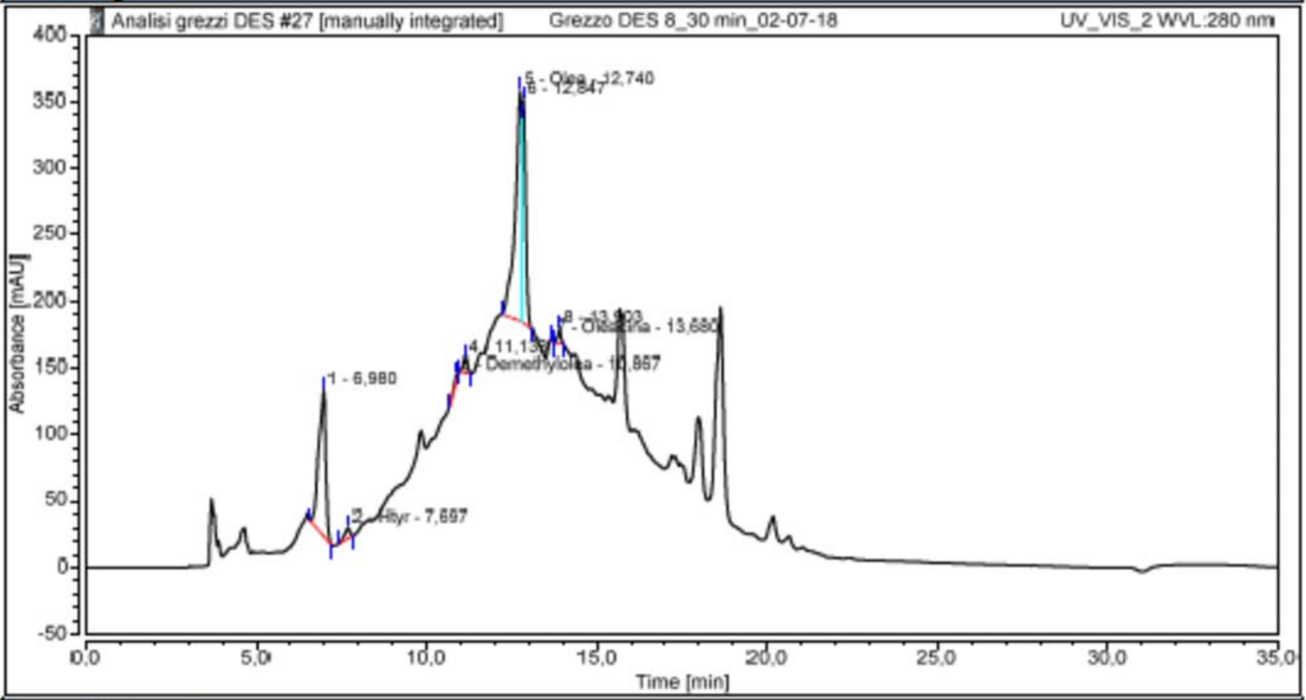

Integration Results

No.	Peak Name	Retention Time min	Area mAU*min	Height mAU	Relative Area %	Relative Height %	Amount ppm
1		6,980	25,791	110,121	29,18	22,48	n.a.
2	Htyr	7,697	1,499	8,400	1,70	1,71	n.a.
3	Demethylolea	10,867	0,697	5,837	0,79	1,19	10,9980
4		11,133	1,542	10,502	1,74	2,14	n.a.
5	Olea	12,740	37,173	173,230	42,06	35,36	22673,6196
6		12,847	20,320	167,536	22,99	34,20	n.a.
7	Oleacina	13,680	0,196	3,270	0,22	0,67	1,6298
8	Aglicone Olea	13,903	1,167	10,946	1,32	2,23	n.a.
Total:			88,385	489,842	100,00	100,00	

Water

Ripe Olives (30 min.)

Chromatogram and Results

Injection Details

Injection Name:	**estratto drupe_2mg/ml_25-01-19**	*Run Time (min):*	**35,00**
Vial Number:	**44**	*Injection Volume:*	**20,00**
Injection Type:	**Unknown**	*Channel:*	**UV_VIS_2**
Calibration Level:		*Wavelength:*	**280**
Instrument Method:	**Oleacina MIX OK**	*Bandwidth:*	**2**
Processing Method:	**Quantificazione Oleacina Mix**	*Dilution Factor:*	**1,0000**
Injection Date/Time:	**25/gen/19 17:59**	*Sample Weight:*	**1,0000**

Chromatogram

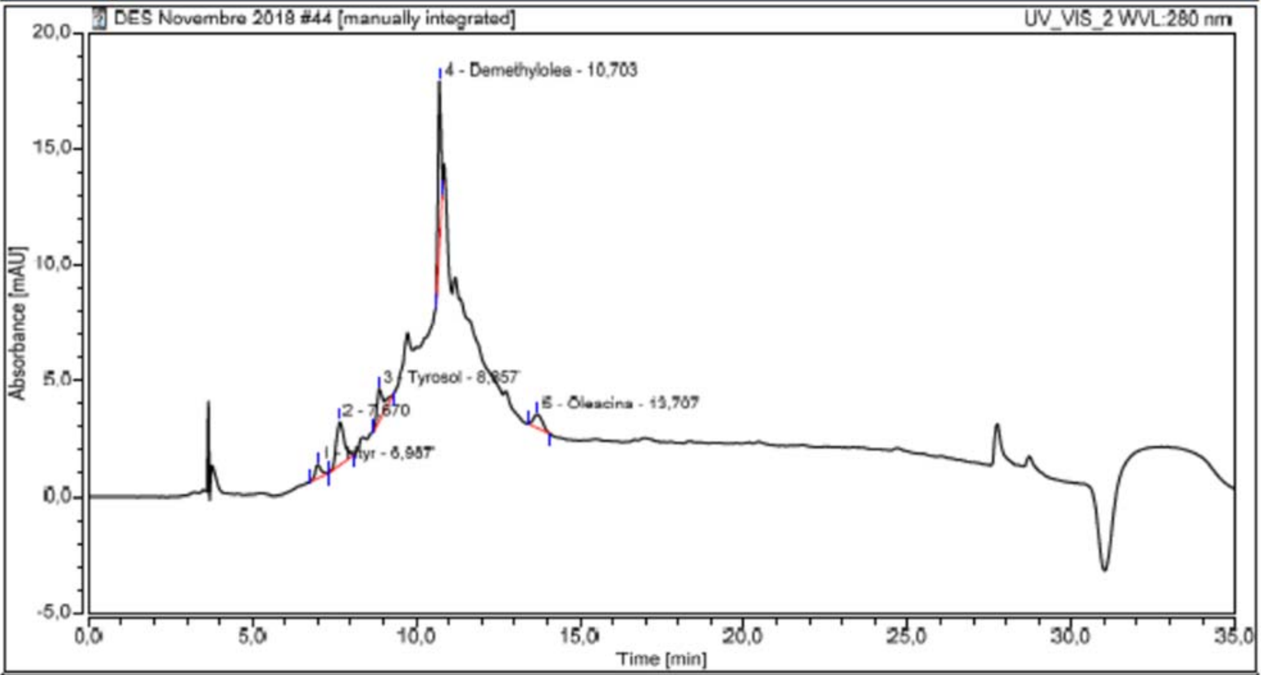

Integration Results

No.	Peak Name	Retention Time min	Area mAU*min	Height mAU	Relative Area %	Relative Height %	Amount ppm
1	Htyr	6,987	0,123	0,599	6,63	5,18	n.a.
2		7,670	0,529	1,884	28,47	16,29	n.a.
3	Tyrosol	8,857	0,309	1,430	16,66	12,36	n.a.
4	Demethylolea	10,703	0,711	7,063	38,27	61,06	11,2053
n.a.	Olea	n.a.	n.a.	n.a.	n.a.	n.a.	n.a.
5	Oleacina	13,707	0,185	0,591	9,98	5,11	1,5665
n.a.	Aglicone Olea	n.a.	n.a.	n.a.	n.a.	n.a.	n.a.
**Total:**			**1,857**	**11,567**	**100,00**	**100,00**	

Table S1: Quantitatives analysis of Oleuropeine in fresh (L.F.) and dried leaves (L.D.) by HPLC

Solvent	values of three analysis L.F. (ppm)			values of three analysis D.F. (ppm)			Mean value (ppm) L.F.	Mean value (ppm) D.F.	SD L.F.	SD D.F.
NADES-1	0	0	0	0	0	0	0	0	0	0
NADES-2	333,54	333,67	333,80	416,25	416,01	415,97	333,73	416,08	0,17	0,15
NADES-3	86,87	85,00	87,02	126,41	126,50	126,61	86,30	126,51	1,12	0,10
NADES-4	0	0	0	73,01	72,98	72,91	0	72,97	0	0,05
NADES-5	0	0	0	0	0	0	0	0	0	0
Water	0	0	0	174,03	174,87	174,51	0	174,47	0	0,42

Table S2: Quantitatives analysis of Oleuropeine in drupes by HPLC

Solvent	values of three analysis (ppm) 10 minutes			values of three analysis (ppm) 30 minutes			Mean value (ppm) 10 min	Mean value (ppm) 30 min	SD 10 min	SD 30 min
NADES-1	0	0	0	0	0	0	0	0	0	0
NADES-2	88312,81	88287,57	88362,33	37601,21	37602,42	37603,63	88320,90	37602,42	38,03	1,21
NADES-3	65976,70	66028,40	66080,10	23328,22	23338,72	23349,22	66028,40	23338,72	51,70	10,50
NADES-4	0	0	0	0	0	0	0	0	0	0
NADES-5	0	0	0	42522,74	42534,86	42546,98	0	42534,86	0	12,12
Water	0	0	0	0	0	0	0	0	0	0

Table S3: Quantitatives analysis of 3,4-DHPEA-EDA in drupes by HPLC

Solvent	values of three analysis (ppm) 10 minutes			values of three analysis (ppm) 30 minutes			Mean value (ppm) 10 min	Mean value (ppm) 30 min	SD 10 min	SD 30 min
NADES-1	0	0	0	0	0	0	0	0	0	0
NADES-2	154,83	156,64	158,46	481,02	480,80	479,98	156,64	480,60	1,81	0,55
NADES-3	0	0	0	194,90	193,15	191,40	0	193,15	0	1,75
NADES-4	418,9901	420,9363	422,82	0	0	0	420,92	0	1,92	0
NADES-5	635,3251	637,0603	638,79	23,85	25,93	27,29	637,06	25,69	1,74	1,73
Water	501,7312	503,05	504,37	780,01	783,25	786,48	503,05	783,25	1,32	3,23

Table S4: Quantitatives analysis of Demethyloleuropein in drupes by HPLC

Solvent	values of three analysis (ppm) 10 minutes			values of three analysis (ppm) 30 minutes			Mean value (ppm) 10 min	Mean value (ppm) 30 min	SD 10 min	SD 30 min
NADES-1	0	0	0	0	0	0	0	0	0	0
NADES-2	283,54	284,67	285,79	1019,31	1019,84	1020,37	284,67	1019,84	1,13	0,53
NADES-3	1916,88	1918,76	1920,64	4551,52	4527,07	4562,62	1918,76	4527,07	1,88	18,19
NADES-4	1653,94	1655,97	1658,00	901,60	903,79	905,91	1655,97	903,77	2,03	2,15
NADES-5	0	0	0	174,01	174,97	175,86	0	174,95	0	0,93
Water	3991,78	4000,55	4009,23	5597,63	5602,65	5607,65	4000,52	5602,65	8,72	5,01

NADES-1-W

Ripe Olives (10 min.)

Chromatogram and Results

Injection Details

Injection Name:	Grezzo DES1W10 min_26-06-18	*Run Time (min):*	35,00
Vial Number:	17	*Injection Volume:*	20,00
Injection Type:	Unknown	*Channel:*	UV_VIS_2
Calibration Level:		*Wavelength:*	280
Instrument Method:	Oleacina MIX OK	*Bandwidth:*	2
Processing Method:	Quantificazione Oleacina Mix	*Dilution Factor:*	1,0000
Injection Date/Time:	26/giu/18 16:03	*Sample Weight:*	1,0000

Chromatogram

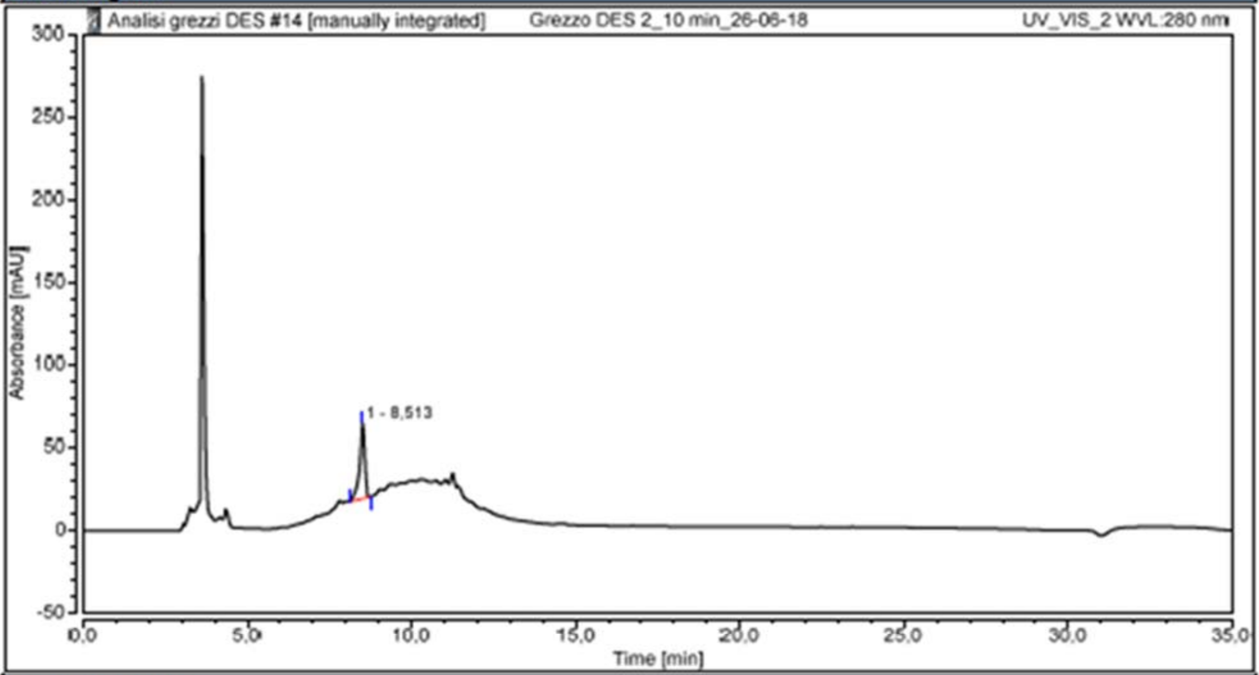

Integration Results

No.	Peak Name	Retention Time min	Area mAU*min	Height mAU	Relative Area %	Relative Height %	Amount ppm
n.a.	Htyr	n.a.	n.a.	n.a.	n.a.	n.a.	n.a.
1		8,513	8,232	45,227	100,00	100,00	n.a.
n.a.	Demethylolea	n.a.	n.a.	n.a.	n.a.	n.a.	n.a.
n.a.	Olea	n.a.	n.a.	n.a.	n.a.	n.a.	n.a.
n.a.	Oleacina	n.a.	n.a.	n.a.	n.a.	n.a.	n.a.
n.a.	Aglicone Olea	n.a.	n.a.	n.a.	n.a.	n.a.	n.a.
Total:			8,232	45,227	100,00	100,00	

NADES-1-W

Ripe Olives (30 min.)

Chromatogram and Results

Injection Details

Injection Name:	Grezzo DES1W30 min_27-06-18	*Run Time (min):*	35,00
Vial Number:	17	*Injection Volume:*	20,00
Injection Type:	Unknown	*Channel:*	UV_VIS_2
Calibration Level:		*Wavelength:*	280
Instrument Method:	Oleacina MIX OK	*Bandwidth:*	2
Processing Method:	Quantificazione Oleacina Mix	*Dilution Factor:*	1,0000
Injection Date/Time:	27/giu/18 10:18	*Sample Weight:*	1,0000

Chromatogram

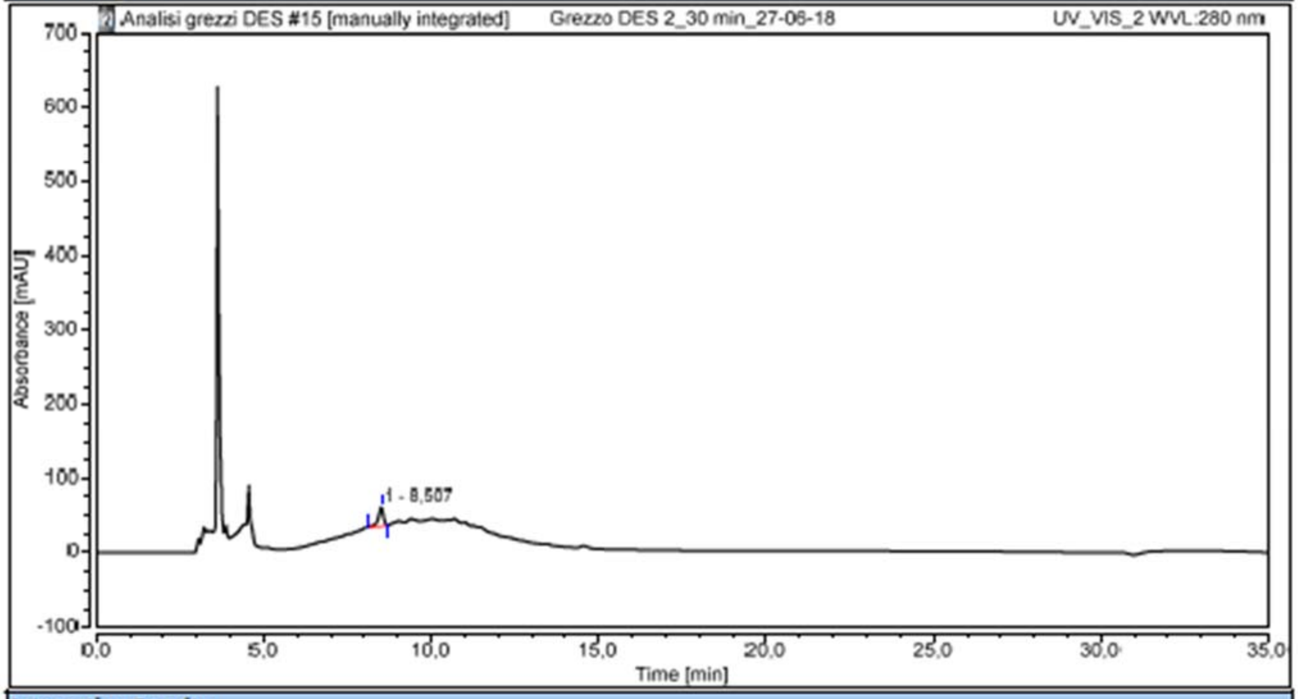

Integration Results

No.	Peak Name	Retention Time min	Area mAU*min	Height mAU	Relative Area %	Relative Height %	Amount ppm
n.a.	Htyr	n.a.	n.a.	n.a.	n.a.	n.a.	n.a.
1		8,507	4,602	26,432	100,00	100,00	n.a.
n.a.	Demethylolea	n.a.	n.a.	n.a.	n.a.	n.a.	n.a.
n.a.	Olea	n.a.	n.a.	n.a.	n.a.	n.a.	n.a.
n.a.	Oleacina	n.a.	n.a.	n.a.	n.a.	n.a.	n.a.
Total:			4,602	26,432	100,00	100,00	

NADES-2-W

Ripe Olives (10 min.)

Chromatogram and Results

Injection Details

Injection Name:	Grezzo DES 2W10 min_27-06-18	*Run Time (min):*	35,00
Vial Number:	17	*Injection Volume:*	20,00
Injection Type:	Unknown	*Channel:*	UV_VIS_2
Calibration Level:		*Wavelength:*	280
Instrument Method:	Oleacina MIX OK	*Bandwidth:*	2
Processing Method:	Quantificazione Oleacina Mix	*Dilution Factor:*	1,0000
Injection Date/Time:	27/giu/18 13:50	*Sample Weight:*	1,0000

Chromatogram

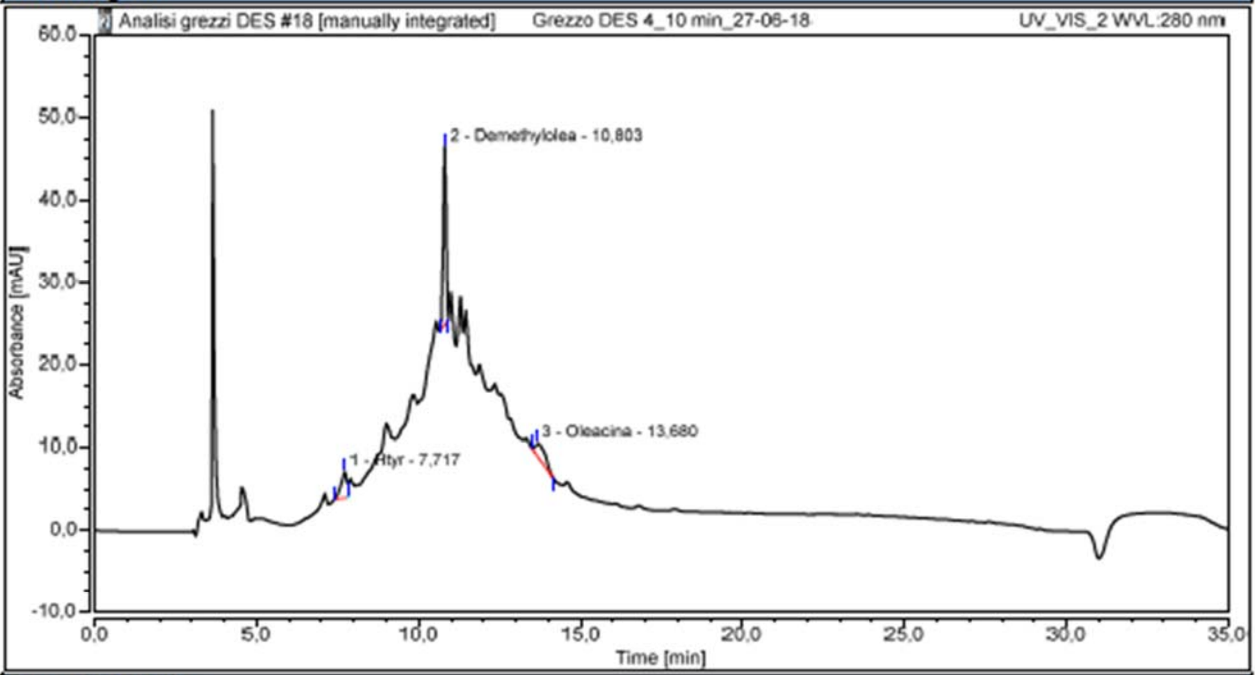

Integration Results

No.	Peak Name	Retention Time min	Area mAU*min	Height mAU	Relative Area %	Relative Height %	Amount ppm
1	Htyr	7,717	0,654	3,187	18,25	12,04	n.a.
2	Demethylolea	10,803	2,278	21,675	63,53	81,88	35,9182
n.a.	Olea	n.a.	n.a.	n.a.	n.a.	n.a.	n.a.
3	Oleacina	13,680	0,653	1,611	18,22	6,08	5,4415
**Total:**			**3,585**	**26,472**	**100,00**	**100,00**	

NADES-2-W

Ripe Olives (30 min.)

Chromatogram and Results

Injection Details

Injection Name:	**Grezzo DES 2w 30 min_27-06-18**	*Run Time (min):*	**35,00**
Vial Number:	**17**	*Injection Volume:*	**20,00**
Injection Type:	**Unknown**	*Channel:*	**UV_VIS_2**
Calibration Level:		*Wavelength:*	**280**
Instrument Method:	**Oleacina MIX OK**	*Bandwidth:*	**2**
Processing Method:	**Quantificazione Oleacina Mix**	*Dilution Factor:*	**1,0000**
Injection Date/Time:	**27/giu/18 15:06**	*Sample Weight:*	**1,0000**

Chromatogram

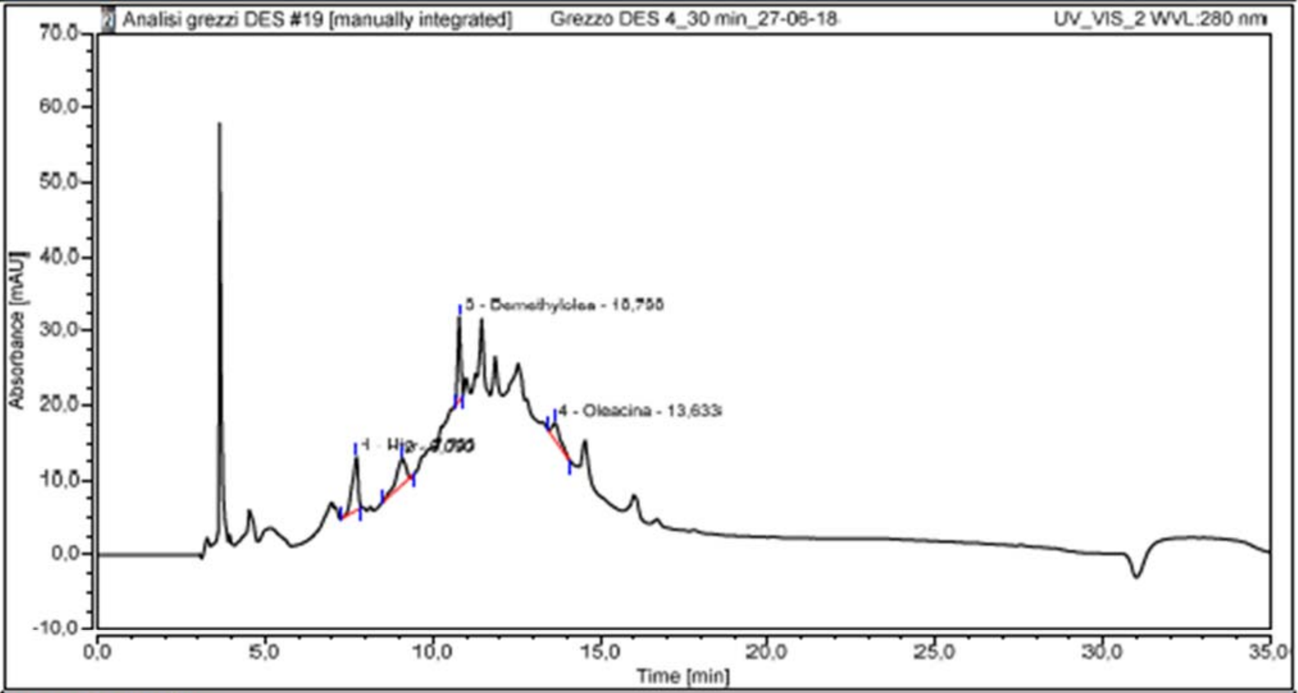

Integration Results

No.	Peak Name	Retention Time min	Area mAU*min	Height mAU	Relative Area %	Relative Height %	Amount ppm
1	Htyr	7,723	1,413	7,082	32,08	29,29	n.a.
2		9,090	1,130	3,423	25,66	14,16	n.a.
3	Demethylolea	10,790	1,198	11,388	27,20	47,10	18,8884
n.a.	Olea	n.a.	n.a.	n.a.	n.a.	n.a.	n.a.
4	Oleacina	13,633	0,663	2,287	15,06	9,46	5,5256
**Total:**			**4,404**	**24,179**	**100,00**	**100,00**	

NADES-3-W

Ripe Olives (10 min.)

Chromatogram and Results

Injection Details

Injection Name:	**Grezzo DES3W10 min_28-06-18**	*Run Time (min):*	**35,00**
Vial Number:	**17**	*Injection Volume:*	**20,00**
Injection Type:	**Unknown**	*Channel:*	**UV_VIS_2**
Calibration Level:		*Wavelength:*	**280**
Instrument Method:	**Oleacina MIX OK**	*Bandwidth:*	**2**
Processing Method:	**Quantificazione Oleacina Mix**	*Dilution Factor:*	**1,0000**
Injection Date/Time:	**28/giu/18 12:52**	*Sample Weight:*	**1,0000**

Chromatogram

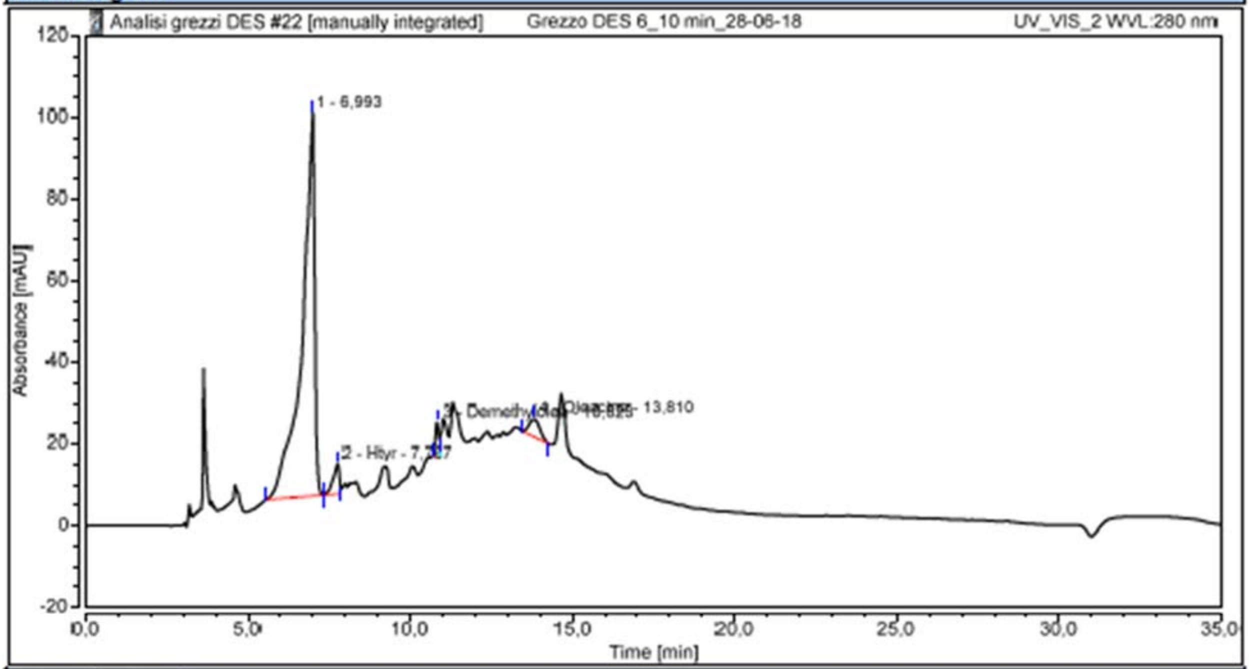

Integration Results

No.	Peak Name	Retention Time min	Area mAU*min	Height mAU	Relative Area %	Relative Height %	Amount ppm
1		6,993	41,376	93,925	90,45	82,27	n.a.
2	Htyr	7,767	1,622	7,457	3,55	6,53	n.a.
3	Demethylolea	10,823	1,069	8,438	2,34	7,39	16,8647
n.a.	Olea	n.a.	n.a.	n.a.	n.a.	n.a.	n.a.
4	Oleacina	13,810	1,676	4,343	3,66	3,80	13,9631
**Total:**			**45,744**	**114,163**	**100,00**	**100,00**	

NADES-3-W

Ripe Olives (30 min.)

Chromatogram and Results

Injection Details

Injection Name:	**Grezzo DES$_{3W}$30 min_28-06-18**	*Run Time (min):*	**35,00**
Vial Number:	**17**	*Injection Volume:*	**20,00**
Injection Type:	**Unknown**	*Channel:*	**UV_VIS_2**
Calibration Level:		*Wavelength:*	**280**
Instrument Method:	**Oleacina MIX OK**	*Bandwidth:*	**2**
Processing Method:	**Quantificazione Oleacina Mix**	*Dilution Factor:*	**1,0000**
Injection Date/Time:	**28/giu/18 14:03**	*Sample Weight:*	**1,0000**

Chromatogram

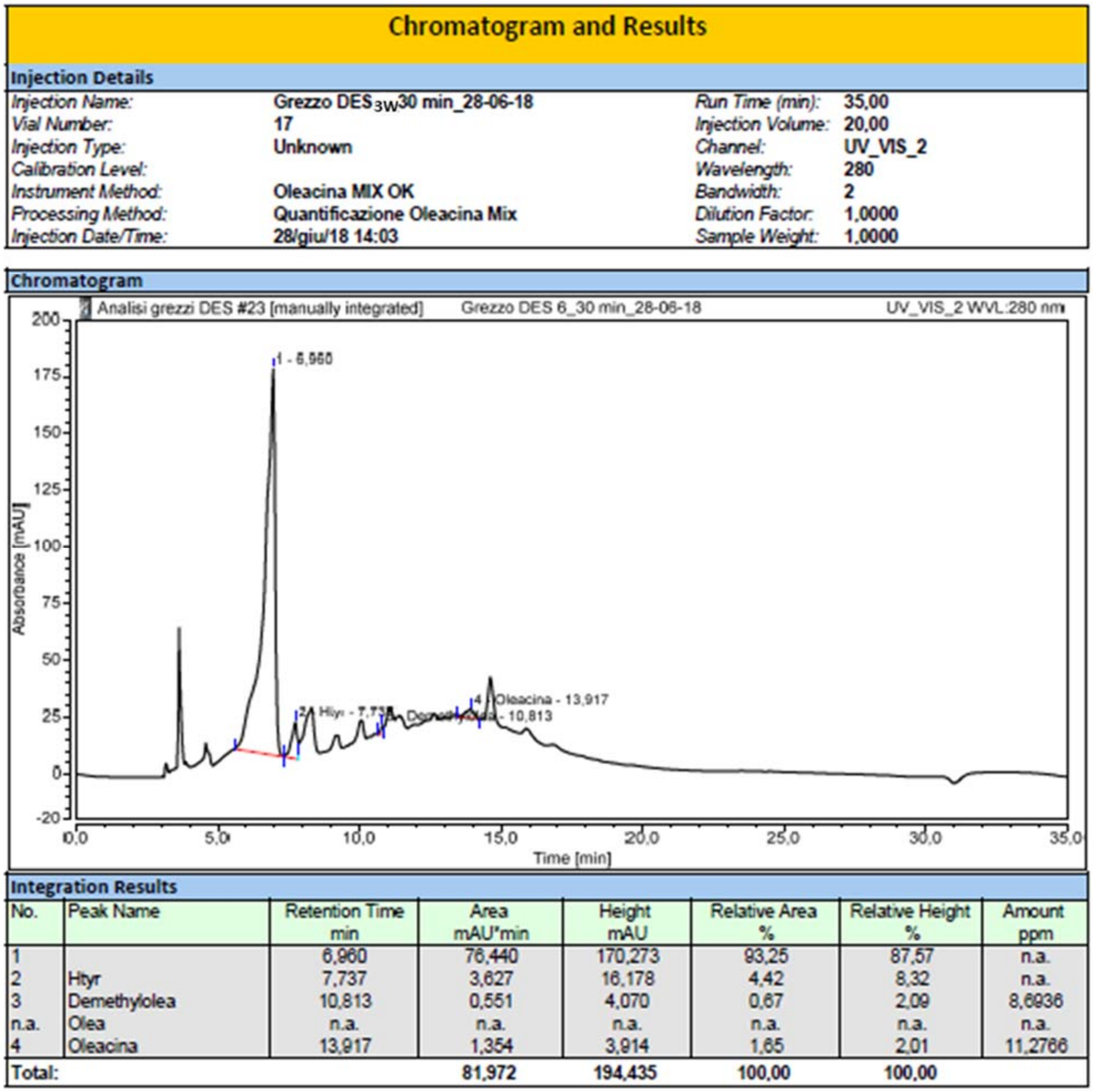

Integration Results

No.	Peak Name	Retention Time min	Area mAU*min	Height mAU	Relative Area %	Relative Height %	Amount ppm
1		6,960	76,440	170,273	93,25	87,57	n.a.
2	Htyr	7,737	3,627	16,178	4,42	8,32	n.a.
3	Demethylolea	10,813	0,551	4,070	0,67	2,09	8,6936
n.a.	Olea	n.a.	n.a.	n.a.	n.a.	n.a.	n.a.
4	Oleacina	13,917	1,354	3,914	1,65	2,01	11,2766
**Total:**			**81,972**	**194,435**	**100,00**	**100,00**	

NADES-5-W

Ripe Olives (10 min.)

Chromatogram and Results

Injection Details

Injection Name:	**Grezzo DES 5W10 min_02-07-18**	*Run Time (min):*	**35,00**
Vial Number:	**11**	*Injection Volume:*	**20,00**
Injection Type:	**Unknown**	*Channel:*	**UV_VIS_2**
Calibration Level:		*Wavelength:*	**280**
Instrument Method:	**Oleacina MIX OK**	*Bandwidth:*	**2**
Processing Method:	**Quantificazione Oleacina Mix**	*Dilution Factor:*	**1,0000**
Injection Date/Time:	**02/lug/18 13:14**	*Sample Weight:*	**1,0000**

Chromatogram

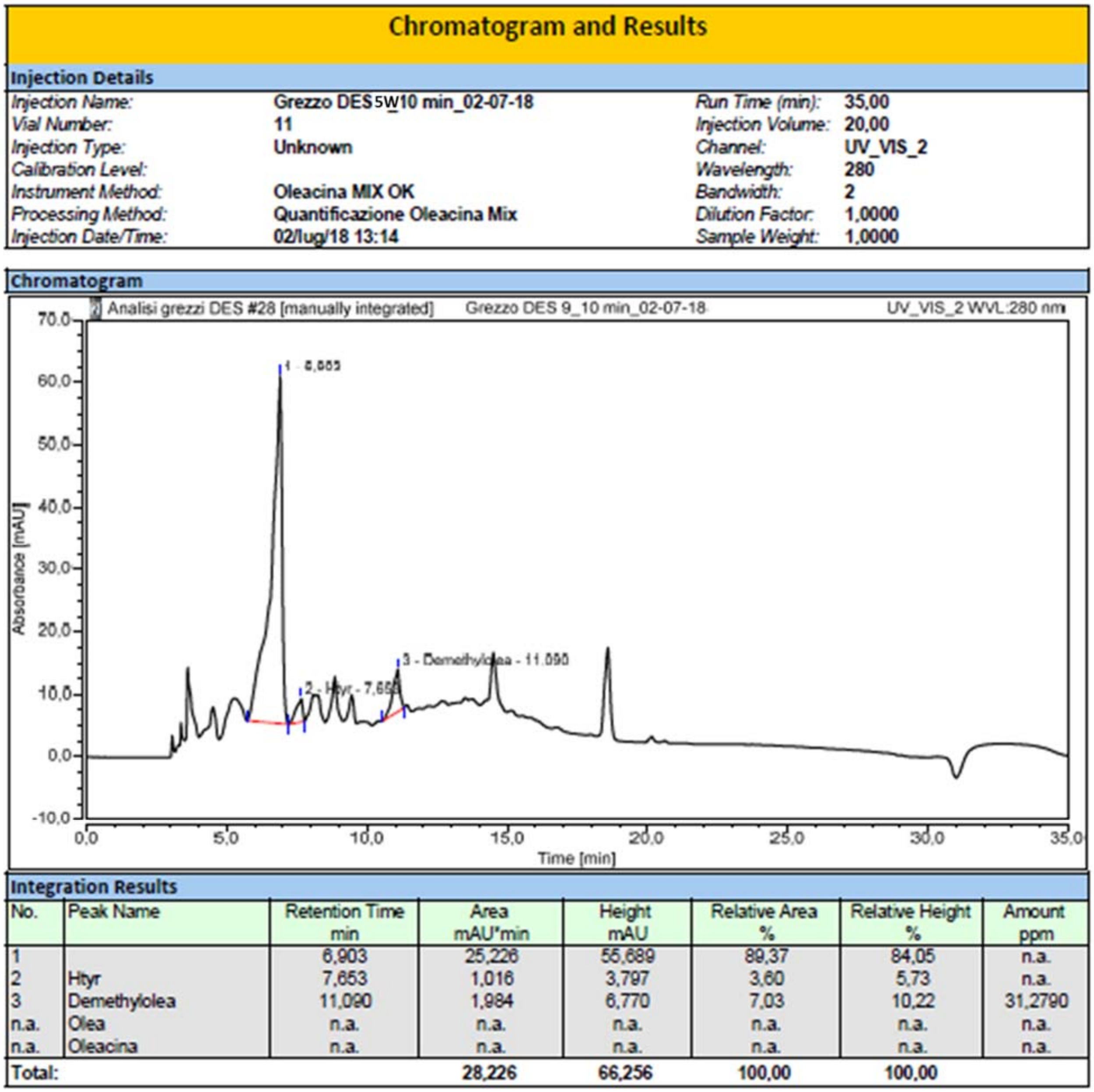

Integration Results

No.	Peak Name	Retention Time<br>min	Area<br>mAU*min	Height<br>mAU	Relative Area<br>%	Relative Height<br>%	Amount<br>ppm
1		6,903	25,226	55,689	89,37	84,05	n.a.
2	Htyr	7,653	1,016	3,797	3,60	5,73	n.a.
3	Demethylolea	11,090	1,984	6,770	7,03	10,22	31,2790
n.a.	Olea	n.a.	n.a.	n.a.	n.a.	n.a.	n.a.
n.a.	Oleacina	n.a.	n.a.	n.a.	n.a.	n.a.	n.a.
**Total:**			**28,226**	**66,256**	**100,00**	**100,00**	

NADES-5-W

Ripe Olives (10 min.)

Chromatogram and Results

Injection Details

Injection Name:	Grezzo DES5W_30 min_02-07-18	*Run Time (min):*	35,00
Vial Number:	13	*Injection Volume:*	20,00
Injection Type:	Unknown	*Channel:*	UV_VIS_2
Calibration Level:		*Wavelength:*	280
Instrument Method:	Oleacina MIX OK	*Bandwidth:*	2
Processing Method:	Quantificazione Oleacina Mix	*Dilution Factor:*	1,0000
Injection Date/Time:	02/lug/18 14:51	*Sample Weight:*	1,0000

Chromatogram

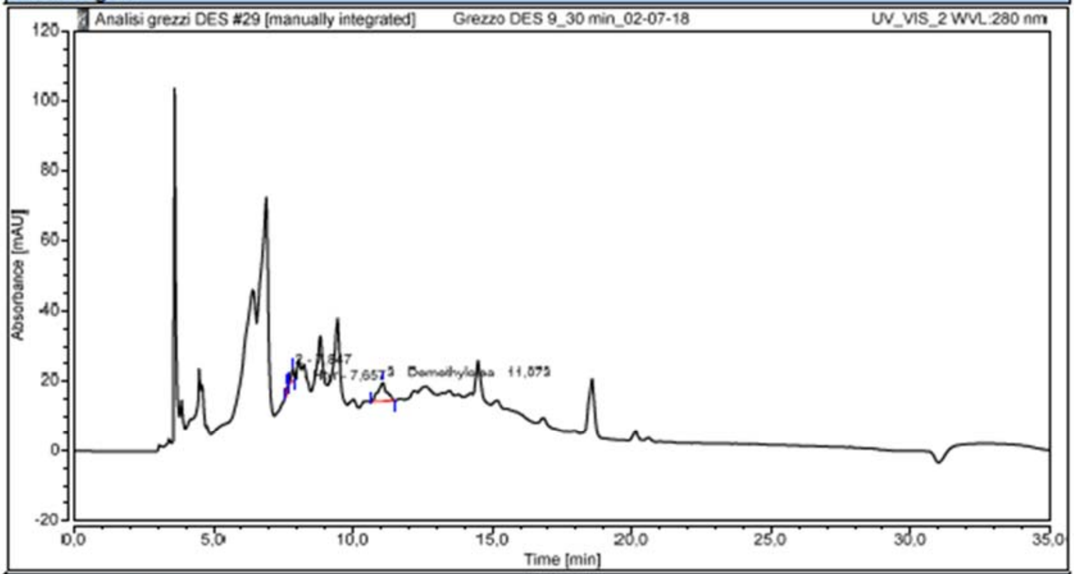

Integration Results

No.	Peak Name	Retention Time min	Area mAU*min	Height mAU	Relative Area %	Relative Height %	Amount ppm
1	Htyr	7,657	0,084	1,104	3,48	10,81	n.a.
2		7,847	0,397	3,614	16,50	35,37	n.a.
3	Demethylolea	11,073	1,926	5,498	80,02	53,82	30,3700
n.a.	Olea	n.a.	n.a.	n.a.	n.a.	n.a.	n.a.
n.a.	Oleacina	n.a.	n.a.	n.a.	n.a.	n.a.	n.a.
**Total:**			**2,407**	**10,216**	**100,00**	**100,00**	
